# Considering Interim Interventions to Control COVID-19 Associated Morbidity and Mortality—Perspectives

**DOI:** 10.3389/fpubh.2020.00444

**Published:** 2020-09-22

**Authors:** Mark Christopher Arokiaraj

**Affiliations:** Department of Cardiology, Pondicherry Institute of Medical Sciences, Kalapet, India

**Keywords:** SARS-CoV-2, influenza vaccination, influenza lower respiratory tract infections, COVID-19 Mortality and morbidity, *Streptococcus pneumonia* vaccine, lower respiratory tract infections

## Abstract

**Aims and objectives:** The pandemic of COVID-19 is evolving worldwide, and it is associated with high mortality and morbidity. There is a growing need to discuss the elements of a coordinated strategy to control the spread and mitigate the severity of COVID-19. H1N1 and *Streptococcus pneumonia* vaccines are available. The current analysis was performed to analyze the severity of COVID-19 and influenza (H1N1) vaccination in adults ≥ 65. Also, to correlate the lower respiratory tract infections (LRIs), and influenza attributable to the lower respiratory tract infections' incidence with Covid-19 mortality. Evolutionarily influenza is close in resemblance to SARS-CoV-2 viruses and shares some common epitopes and mechanisms.

**Methods:** Recent influenza vaccination data of 34 countries from OECD and other publications were correlated with COVID-19 mortality from worldometer data. LRIs attributable to influenza and streptococcus pneumonia were correlated with COVID-19 mortality. Specifically, influenza-attributable LRI incidence data of various countries (*n* = 182) was correlated with COVID-19 death by linear regression and receiver operating characteristic (ROC) curve analyzes. In a logistic regression model, population density and influenza LRI incidence were correlated with COVID-19 mortality.

**Results:** There is a correlation between COVID-19-related mortality, morbidity, and case incidence and the status of influenza vaccination, which appears protective. The tendency of correlation is increasingly highlighted as the pandemic is evolving. In countries where influenza immunization is less common, there is a correlation between LRIs and influenza attributable to LRI incidence and COVID-19 severity, which is beneficial. ROC curve showed an area under the curve of 0.86 (CI 0.78 to 0.944, *P* < 0.0001) to predict COVID-19 mortality >150/million and a decreasing trend of influenza LRI episodes. To predict COVID-19 mortality of >200/million population, the odds ratio for influenza incidence/100,000 was −1.86 (CI −2.75 to −0.96, *P* < 0.0001). To predict the parameter Covid-19 mortality/influenza LRI episodes^*^1000>1000, the influenza parameter had an odd's ratio of −3.83 (CI −5.98 to −1.67), and an AUC of 0.94.

**Conclusion:** Influenza (H1N1) vaccination can be used as an interim measure to mitigate the severity of COVID-19 in the general population. In appropriate high-risk circumstances, *Streptococcus pneumonia* vaccination would also be an adjunct strategy, especially in countries with a lower incidence of LRIs.

## Introduction

As the COVID-19 pandemic is evolving worldwide, the need for a working therapy or method of control is significant to lessen the severity and mortality of coronavirus disease-2019 (COVID-19). Among the influenza viruses, respiratory syncytial viruses are common etiologies for lower respiratory tract infections. *Streptococcus pneumonia* and Hemophilus influenza are common bacterial infections, and *Streptococcus pneumonia* constitutes a significant share of lower respiratory infections. Influenza (H1N1) and *Streptococcus pneumonia* vaccines are commonly available.

The influenza viruses and SARS-CoV-2 viruses have evolutionary proximity. The influenza and coronaviruses utilize similar and contrasting approaches to control interferon-stimulated gene responses (1). Also, the pathognomonic spike protein shares common features with class 1 viral membrane fusion proteins, including influenza viruses (2, 3). The cell entry of the influenza A (H7N9) viruses is through ACE-2 receptors in the lung (4). The exact receptor mechanism of the virus entry is still not clear, however, the surface hemagglutinin receptor binding sites attaches the virus to surface glycoconjugates that contain terminal sialic acid residues (5, 6). H1N1 infections can downregulate the ACE-2 levels in the lung tissues by neuraminidase (7). Hence, the study was performed to analyze the severity of COVID-19 and influenza vaccination in adults >65 years. Since this vaccination is not mandatory for routine clinical practice, various countries adopt different policies, and the vaccination rates significantly differ among various countries.

For further primary prevention, a *Streptococcus pneumonia* vaccine would be a useful strategy that is known to be effective (8–10). Bacterial infections are commonly associated with viral pneumonia (11). The addition of a *Streptococcus pneumonia* vaccine can further improve the protective benefits by an additional or logarithmical value, which is yet to be determined.

## Methods

The influenza vaccination status data in the elderly ≥ 65 years was obtained from OECD (organization for economic cooperation and development) data (12). The data of countries that are not available in the OECD data were obtained from various publications (13, 14)^1^, (15, 16). At the time of this writing, the details of the COVID-19 were obtained from worldometer data/coronavirus, and the current mortality worldwide is 725,000. The number of critical or severe patients were obtained from the same source. The critical illness data was compared with the respective population of the countries.

The incidence, mortality, and case fatality rate of COVID-19 were correlated with influenza vaccination status in adults ≥ 65 years. The tests performed vary in different countries, and the clinical parameters vary based on the tests performed. Hence, some of the results were corrected for the tests performed. Subsequently, closed cohorts of patients were studied and analyzed for COVID-19 mortality and influenza vaccination status. The closed cohorts included US military and veteran's affairs, US healthcare professionals, and cruise ship data. The herd immunity of COVID-19 reported in various papers was studied to analyze the necessity and significance of the influenza vaccination in the current COVID-19 situation. To envisage the possible mechanisms of the influenza vaccination and its protective benefit against COVID-19 severity, the transcriptomic profile and T/B-cell responses and the overlapping features of influenza A viruses and COVID-19 were studied, taking basis in various published articles.

To further analyze the significance of influenza LRI's burden all over the world's and its correlation with COVID-19 mortality, the lower respiratory tract infections data were analyzed with COVID-19 death through global disease burden (GBD) data. The influenza-attributable LRI details were analyzed through historical GBD data—the influenza LRI incidence was correlated with the current COVID-19 worldometer data. When a significant correlation was observed, receiver operating statistic curve analyses were performed in two separate time occasions. After that, the Influenza LRI data (x) was correlated with Covid19 mortality (y) using the x and y/x method. The receiver operating characteristic (ROC) curve analyses was performed with COVID-19 mortality cut-off values and decreasing incidence of influenza LRI episodes. In a logistic regression model with influenza LRI and population density parameters, the COVID-19 mortality was studied.

## Results

### COVID-19 Case-Fatality Rate and Influenza LRI Parameter

Logistic regression analysis shows the influenza parameter is consistently associated with the Covid19 case-fatality rate, i.e., mortality/cases. In a logistic regression model including the parameters influenza LRI incidence*population density, population density, population in numbers, and influenza LRI incidence parameter, the lesser values of influenza LRI parameter was consistently associated with Covid19 mortality/cases rate (Supplement Figure S17). To indicate the Covid19 mortality/cases rate >5 percent the odds ratio for influenza LRI parameter was −0.681 (CI −1.147 to −0.214, *P* = 0.004, August 23, 2020), for rate >4 the odds ratio was −0.47 (CI −0.823 to −0.118, *P* = 0.009) and for >3 the odds ratio was −0.296 (CI −0.543 to −0.05, *P* = 0.019), and the regression models were significant, as reflected by the respective ROC curves. Individually, the odds ratio for influenza LRI parameter (Supplement Figure S18) associated with COVID-19 mortality/cases rate >3 percent was −0.206 (−0.403 to 0.008, *P* = 0.041), for rate >4 the odds ratio was −0.419 (CI −0.715 to −0.122, *P* = 0.006), and for case fatality rate >5 the value was −0.662 (−1.055 to −0.269, *P* = 0.001). Influenza*Population density had an odds ratio of −0.402 (−1.01 to +0.2, *P* = 0.196), and though it was not statistically significant, it tended to be associated with high case-fatality rate (Supplement Figure S19, Panel A).

The latest odd ratio values (August 23, 2020) for influenza LRI *population density parameter to indicate Covid19 parameter >250/million (Supplement Figure S19 panel B) was −2.73 (−5.12 to −0.34, *P* = 0.025) and the odds ratio for influenza parameter to indicate Covid19 mortality >250/million (Supplement Figure S19 panel C) was −0.913 (−1.42 to −0.406, *P* = 0.000).

### COVID-19 Incidence

**Figure 1:**
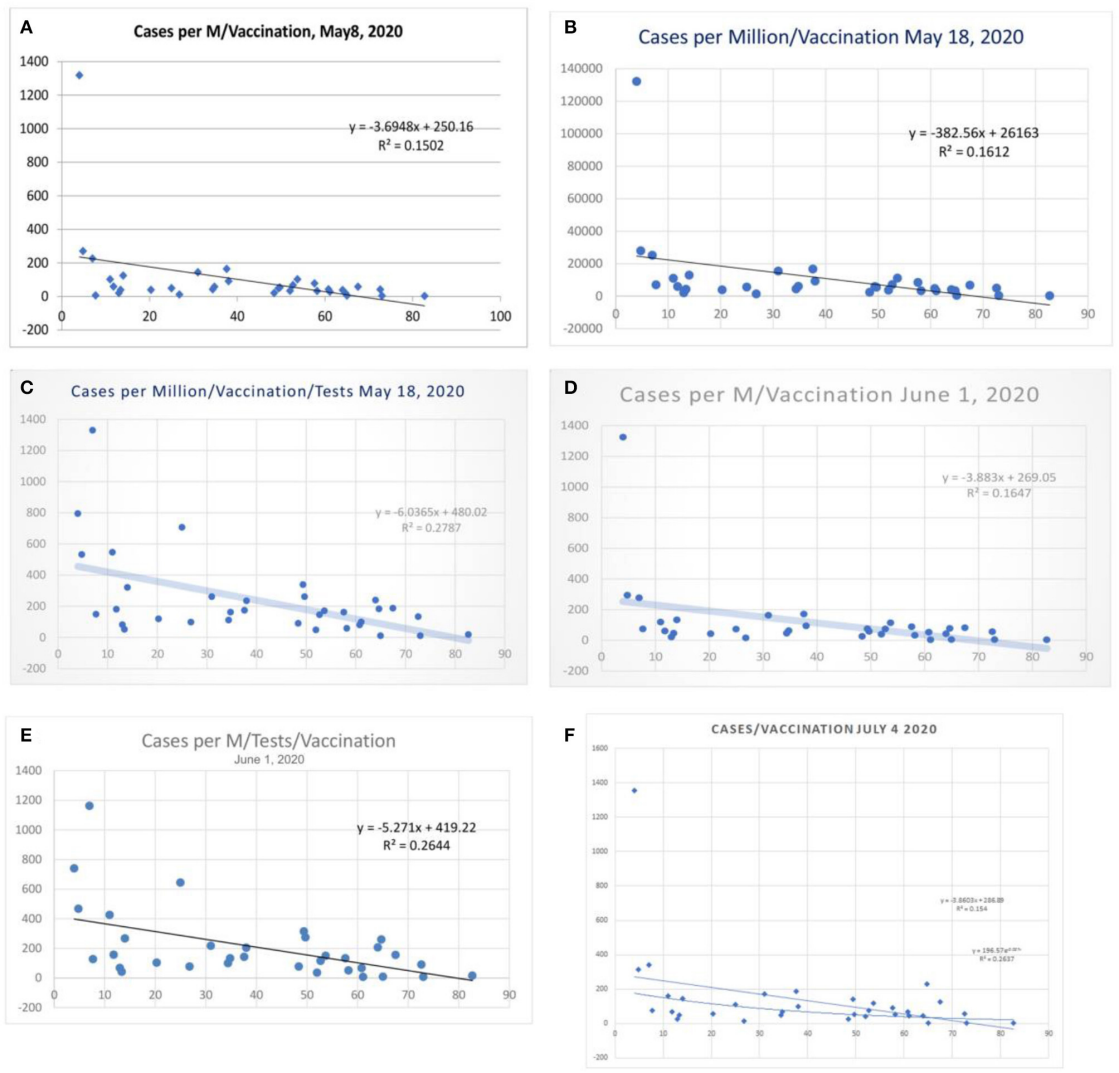
Correlation between influenza vaccination percentage (age ≥ 65 years), and (cases per million population) vaccination percentage, **(A)** May 8, 2020, and **(B)** May 18, 2020. **(C)** Shows the case per-million value adjusted to tests performed (May 18, 2020) and **(D,E)** the data of June 1 2020. **(F)** Shows linear and logarithmic trendlines (July 4, 2020).

Table 1 shows the statistical data from the worldometer data/coronavirus at the time of this write-up. The correlation between vaccination and cases per million/vaccination shows an *R*^2^ of 0.16 and a variance benefit of 15% (Figures 1A–F). When adjusted to the tests performed that modulate the case identification, the benefit is more pronounced, and *R*^2^ was 0.28 (*r* = −0.53, Figure 1C) and is sustained around 0.26 (Figure 1E), and the latest *R*^2^ was 0.15 (Panel F lower line, Logarithmic *R*^2^ 0.26). There is a tendency toward a lesser case incidence in vaccinated countries.

**Table 1:** Incidence of mortality and critical position of the patient and influenza vaccination status (age ≥ 65 years) in different countries (April 10, 2020).

**Country**	**Mortality/million population**	**Latest influenza vaccination statistics (%)**	**Critical/serious numbers (*n*)**	**Population in million**	**Critical numbers/population in M**
USA	50	67.5	10,011	330	60.1
Spain	330	53.7	7,371	47	59.7
Italy	302	52.7	3,605	60	60.1
Germany	31	34.8	4,895	82	59.7
France	187	49.7	7,066	67	105.5
Iran	49	25	3,987	66	60.4
UK	118	72.6	1,559	81	19.2
Chile	3	64.7	360	18	20
Belgium	218	31	1,285	11.5	111.7
Switzerland	110	38	386	8	48.2
Netherlands	140	64	1,424	17.3	82.3
Canada	13	61.1	518	37.6	13.8
Austria	33	14	266	9	29.6
Portugal	40	60.8	241	10.3	23.3
South Korea	4	82.7	55	51.5	1.07
Sweden	79	49.4	719	10.2	70.4
Norway	20	34.4	82	5.4	15.1
Finland	8	48.4	78	5.5	14.1
Denmark	41	52	160	5.5	29.1
Luxembourg	83	37.6	30	0.6	50
Estonia	18	4.8	9	1.4	6.4
Iceland	18	45	11	0.37	29.7
Australia	2	73	81	24	3.3
New-Zealand	0.4	65	4	4.8	0.83
Ireland	53	57.6	165	4.9	33.7
Hungary	7	26.8	17	9.7	1.75
Israel	10	58.2	166	8.7	19.1
Lithuania	6	13.4	21	2.8	7.5
Czech Republic	10	20.3	96	10.5	9.1
Latvia	2	7.7	3	2	1.5
Serbia	8	11	112	7	16
Slovak republic	0.4	13	3	5.4	0.55
Turkey	11	7	1,552	82	18.9
Slovenia	21	11.8	34	2.1	16.2

### COVID-19 Mortality and Influenza Vaccination Status

**Figure 2:**
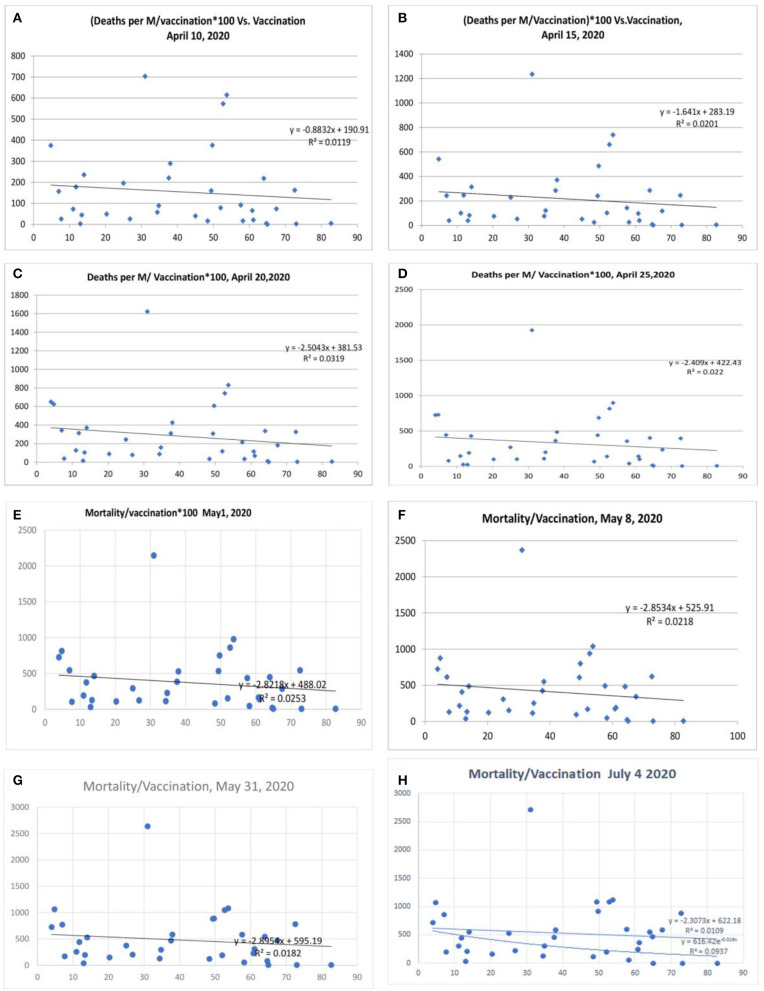
Correlation between influenza vaccination percentage (age ≥ 65 years) and mortality per million population/vaccination percentage [**(A)** April 10, 2020, **(B)** April 15, 2020, lower panel April 20, 2020, **(C)** April 20, 2020, **(D)** April 25, 2020, **(E)** May 1, 2020, **(F)** May 8, 2020, **(G)** May 31, 2020, and **(H)** July 4, 2020].

**Figure 3:**
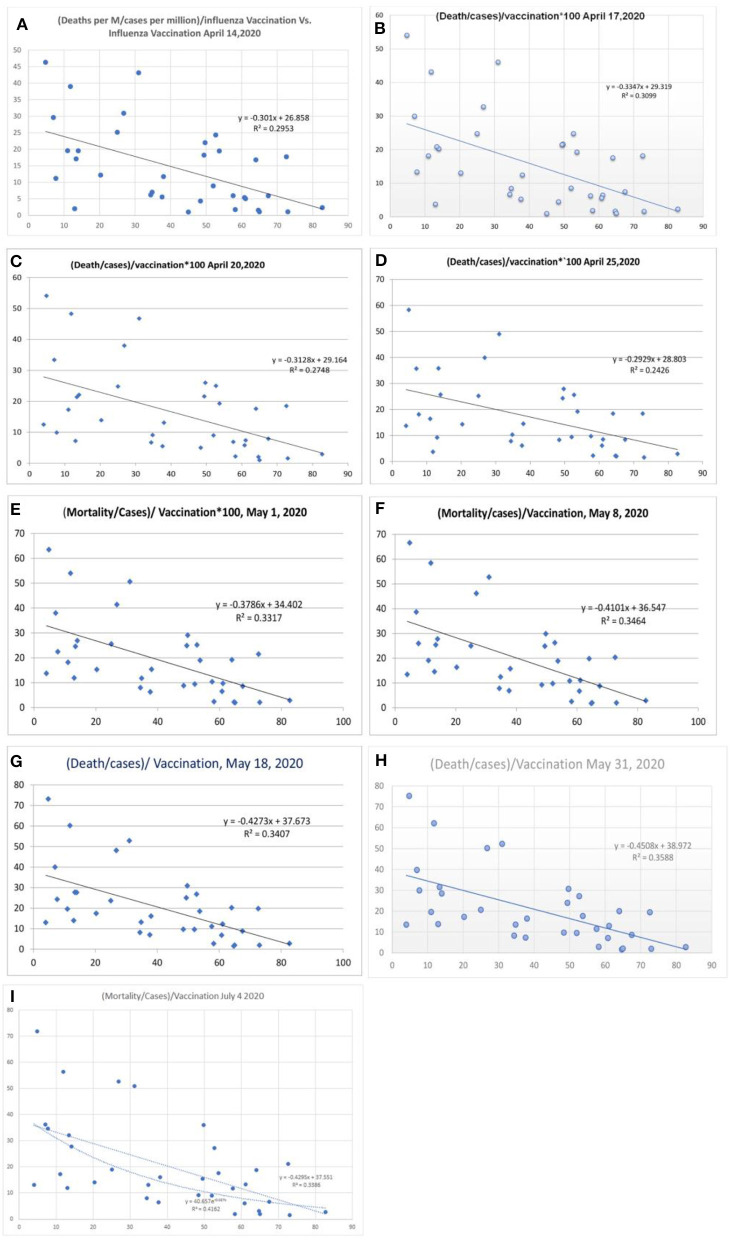
Correlation between influenza vaccination percentage vs. [(deaths/million)/cases per million]/vaccination [**(A)** April 14, 2020, **(B)** April 17, 2020, **(C)** April 20, 2020, **(D)** April 25, 2020, **(E)** May 1, 2020, **(F)** May 8, 2020, and **(G)** May 18, 2020, **(H)** May 31, 2020, and **(I)** July 4, 2020].

**Figure 4:**
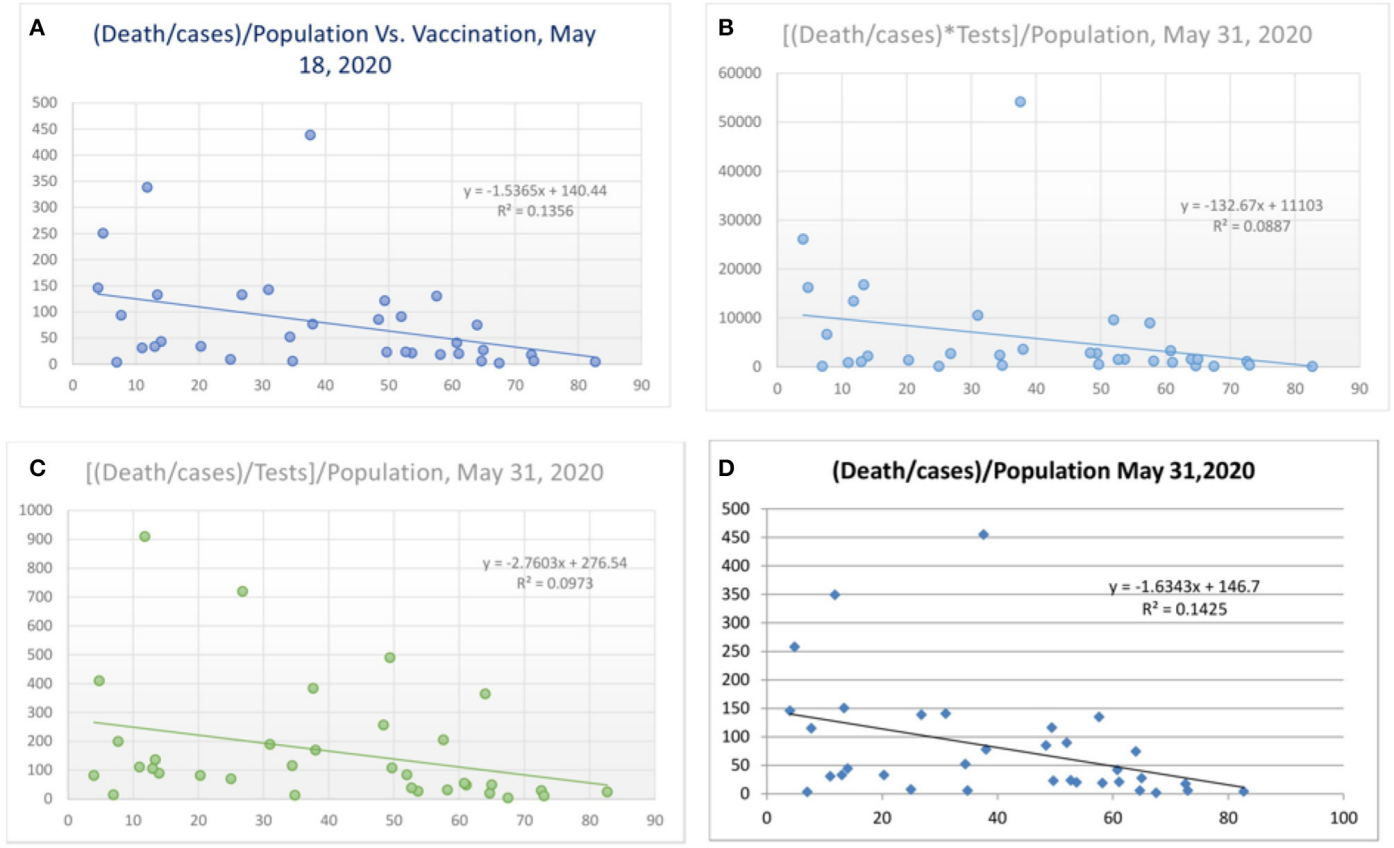
Correlation between vaccination and death per million/cases per million adjusted to population [**(A)** May 18, 2020 and **(D)** May 31, 2020] and tests performed [**(B)** and **(C)**, May 31, 2020].

There was a statistical correlation between the influenza vaccination status and the mortality of the COVID-19 illness (Figures 2A–H). The latest *R*^2^ was 0.01 and logarithmic *R*^2^ was 0.0937 (Figure 2H). The correlation graph shows vaccination status and mortality reduction. When the mortality figures were adjusted to the case numbers/million population, the mortality benefit was significant (Figures 3A–I). Correlation graphs showed a variance of about 25–34.6% toward mortality benefit. The latest *R*^2^ value of July 4, 2020, was 0.338 (Figure 3I, upper line), and the logarithmic *R*^2^ was 0.416. Though it is not a traditional *R*^2^ > 0.7, there are many parameters affecting mortality. Since mortality is a major end point, and it can be affected by various parameters pertaining to baseline characteristics and clinical and demographic variations, the variance benefits from immunization alone are significant. Hence, the results in Figure 3 correlation have a contribution to mortality reduction. A correlation between vaccination and mortality/cases per-million adjusted to population (Figures 4A,D) showed an *R*^2^ of 0.13 (*r* = −0.367) to 0.14. When it was further adjusted to the tests performed, the *R*^2^ was about 0.09 to 0.1 (Figures 4B,C).

### Corrections for Tests Performed

**Figure 5:**
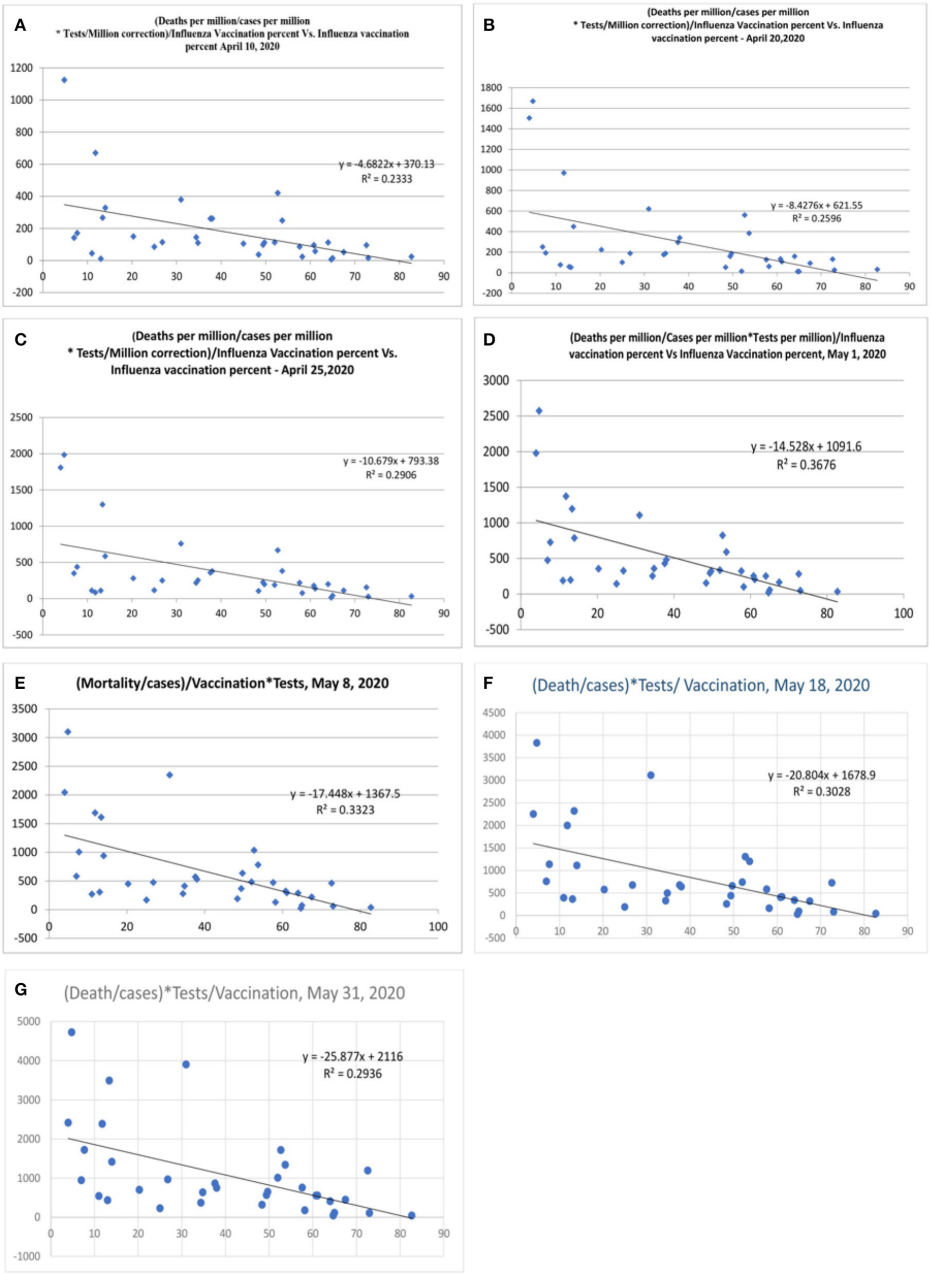
Correlation between influenza vaccination percentage (age ≥ 65 years), vs. [(Deaths/million)/Cases per million]/Vaccination after correction for tests performed per million [**(A)**, April 10, 2020, **(B)** April 20, 2020, **(C)** April 25, 2020, **(D)** May 1, 2020, **(E)** May 8, 2020, **(F)** May 18, 2020, and **(G)** May 31, 2020].

**Figure 6:**
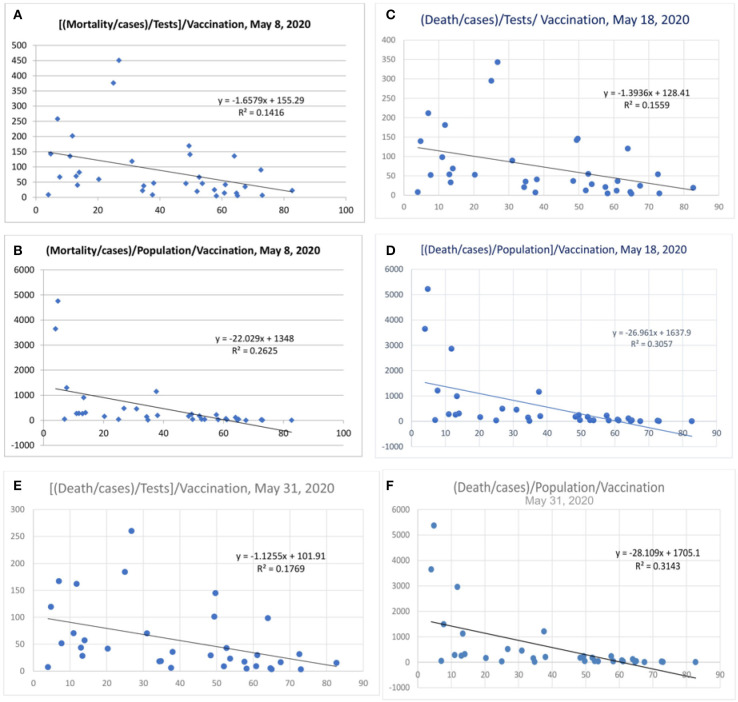
Mortality/Cases adjusted to tests by denominator **(A,C,E)**. Mortality/cases values adjusted to population in **(B,D,F)**.

Some countries have differences in their testing strategy, and the results were therefore further adjusted with tests performed/million population. The correlation was performed after correction for tests performed/million people (Figure 5 and Table 2). The vaccination parameter after correction for tests performed/million showed a variance of 23.3% (*r*^2^, Figure 5A; April 15, 2020), which increased to 25.9% (Figure 5B; April 20, 2020), and thereafter to 29.1 (Figure 5C; April 25, 2020), and 36.7% (Figure 5D, May 1, 2020); and the last values were 33% (Figure 5E, May 8, 2020), 30% (*r* = −0.55, Figure 5F, May 18, 2020) and 29% (Figure 5G, May 31, 2020). There is also a tendency for more correlation as the pandemic is evolving. When the tests performed were used as a devisor instead of multiplication, the R-value was about 0.16 (*r* = −0.4, Figure 6) with the latest being 0.176 (May 31, 2020—Figure 6). The multiplication technique reflects the magnitude of the problem and the effect of vaccination. The devisor technique demonstrates the impact of the quality of care and the modulation of immunization on mortality. Therefore, both methods are useful for analysis. When the death per million/cases per million parameters was adjusted to the populations of concerned countries, the *R*^2^-value ranged from 0.26 (*r* = −0.51) to 0.3 (*r* = −0.55).

**Table 2:** Death/million population and tests performed/million population (Data April 14, 2020).

**Country**	**Latest influenza vaccination statistics**	**Deaths in M/cases in M population**	(Deaths/cases) *100/influenza vaccination	**Tests/M**
USA	67.5	4.0	5.9	8.8
Spain	53.7	10.5	19.5	12.8
Italy	52.7	12.8	24.3	17.3
Germany	34.8	2.4	7.0	15.7
France	49.7	10.9	22.0	5.1
Iran	25	6.3	25.1	3.4
UK	72.6	12.9	17.7	5.4
Chile	64.7	1.0	1.6	4.4
Belgium	31	13.4	43.1	8.8
Switzerland	38	4.5	11.7	22.3
Netherlands	64	10.8	16.8	6.7
Canada	61.1	3.1	5.1	11.5
Austria	14	2.7	19.5	16.8
Portugal	60.8	3.3	5.4	17.9
South Korea	82.7	1.9	2.3	10.2
Sweden	49.4	9.0	18.2	5.4
Norway	34.4	2.1	6.2	23.4
Finland	48.4	2.1	4.3	8.5
Denmark	52	4.6	8.9	12.7
Luxembourg	37.6	2.1	5.6	46.8
Estonia	4.8	2.2	46.3	24.3
Iceland	45	0.45	1.0	103.3
Australia	73	0.8	1.1	14.3
New-Zealand	65	0.7	1.1	13.5
Ireland	57.6	3.4	6.0	14.5
Hungary	26.8	8.3	30.9	3.7
Israel	58.2	1.0	1.8	13.5
Lithuania	13.4	2.3	17.1	15.6
Czech Republic	20.3	2.5	12.2	12.3
Latvia	7.7	0.9	11.2	15.3
Serbia	11	2.2	19.6	2.3
Slovak republic	13	0.26	2.0	5.5
Turkey	7	2.1	29.6	4.8
Slovenia	11.8	4.6	39.0	17.2

### Influenza, Lower Respiratory Tract Infections, and COVID-19 Mortality

There were few countries with a small population that did not show a correlation. A search was performed to analyze the incidence of influenza in countries that did not show a correlation. Among the countries studied, Latvia, Serbia, Slovak, Turkey, Slovenia, Czech Republic, and Slovenia were the countries that showed no correlation with vaccine status. In these countries, the incidence if flu was greater and perennial, i.e., throughout the year, as opposed to the seasonal pattern seen in other countries like Spain, Italy, Belgium, the Netherlands, Sweden, Scotland, and Luxembourg^2^. The incidence of influenza in Turkey and neighboring countries was high^3^. Also, different countries are in the various stages of disease presentation and spread, and they can hence differ in mortality statistics.

The Central and Eastern European and Central Asian countries have a high incidence of lower respiratory tract infections (LRIs), which are 41.4/1000, 81.2/1000, and 58.5/1000 compared to the high-income countries of North America (38.8/1000) and Western Europe (28.7/1000) (17). In the elderly population, the statistical numbers for LRI are 120/1000, 203/1000, and 168.5/1000 for Central and Eastern European and Central Asian countries, respectively, when compared to the high-income 73.2/1000 in North America and 101/1000 in Western Europe. Analyzing the data of children (<5 years) who play an essential role in herd immunity, the high-income nations have an LRI incidence of about 44.6/1000, Central and Eastern European counties have 107/1000, and Asian countries have an incidence of 120/1000. Hence, in the Eastern European and Central Asian countries have higher LRI episodes and fewer COVID-19-related deaths. This includes Russia, which is classified under Eastern European countries. Similarly, Turkey, which is classified under North African and Middle Eastern countries, has overall LRI episodes of 56.5/1000, and the incidence is 133.2/1000 in the population of children and 246/1000 in the elderly (17).

South Korea has the highest vaccination rates of 82.7% in elderly vaccination (>65 years) and had an early incidence of the pandemic, has the least mortality compared to the early onset countries (worldometer data). Some of South Korea's past data showed influenza vaccination rates up to 86% in the elderly (>65 years) (18). In the US, adult (>18 years) vaccination rates were higher in the states of North Carolina, Washington, Iowa, Maryland, Connecticut, and the Massachusetts areas, with a mean vaccination rate of 52%^4^. The mortality burden is lower in these states compared to other states within US, where the mean vaccination rate was about 47%. Neighboring countries with similar climatic conditions like Belgium and the Netherlands have variations in mortality, i.e., 847/million and 358/million, respectively. The vaccination status in the elderly (>65 years) differs between Belgium and the Netherlands at ~31 and 64%, respectively.

### Closed Cohorts—US Military, Diamond Princess, Ruby Princess

Data from the US military cohort shows an incidence of coronavirus cases of 36,659 cases, and the mortality is at 56 cases to date. The case-fatality ratio is 0.15%, which is the least (July 25, 2020). Age distribution of the mortality statistics is not available for further analysis. The military personnel are known for regular vaccination schedules, including influenza, *Streptococcus pneumonia*, and Hemophilus influenza, and this can represent useful data (19). Better nutrition and improved physical health also add to this outcome. US veteran COVID-19 worldometer data show a mortality rate of 1,999 out of 36,480 cases with a case fatality of 5.5%. However, advanced age and more comorbidities like diabetes are associated, and the rate influenza vaccination was about 71–75%, extending to 82% in some subgroups (20).

The cruise ship Diamond Princess represents another closed cohort, and the vaccination details are not available. Nevertheless, the average vaccination statistics can be assumed to be 50%; the average adult (>18 years) US data for vaccination is 48%, and Scotland has an average of 50% (21). The total members on board including crew was 3,711. A total of 712 cases of COVID-19 were reported, the mortality is about 13 cases to date, and seven patients are serious or critically ill (22). However, a head-to-head comparison is not feasible as the population of the Diamond Princess who were > 70 age was 1,230 out of the total 3,711, and the mean age was 58 years. The case-fatality ratio is 1.8%; however, it could be higher as the details of patients repatriated to various countries after dis-embarkment is not available. The other cruise ship, Ruby Princess, had an incidence of 686, and the mortality number was 21 patients to date (23). The case-fatality ratio was 3%. Other cruise ships like MS Zaandam (Holland-America) reported fewer case numbers, and it was consequently not taken in for analysis.

### Healthcare Professionals—Vaccination and COVID-19 Mortality

In the US, among the healthcare workers, the influenza vaccination rates are about 81%. In particular, physicians (96.7%) and nurses (98.1%) have very high vaccine uptake rates^5^. Among other allied healthcare professionals, however, the vaccine uptake rate ranges from 75 to 85%. So far, the incidence among healthcare workers has been 113,730, and the mortality was 576 cases (July 25, 2020), which is 0.5% (<1%)^6^.

### COVID-19—Critical Illness Numbers

**Figure 7:**
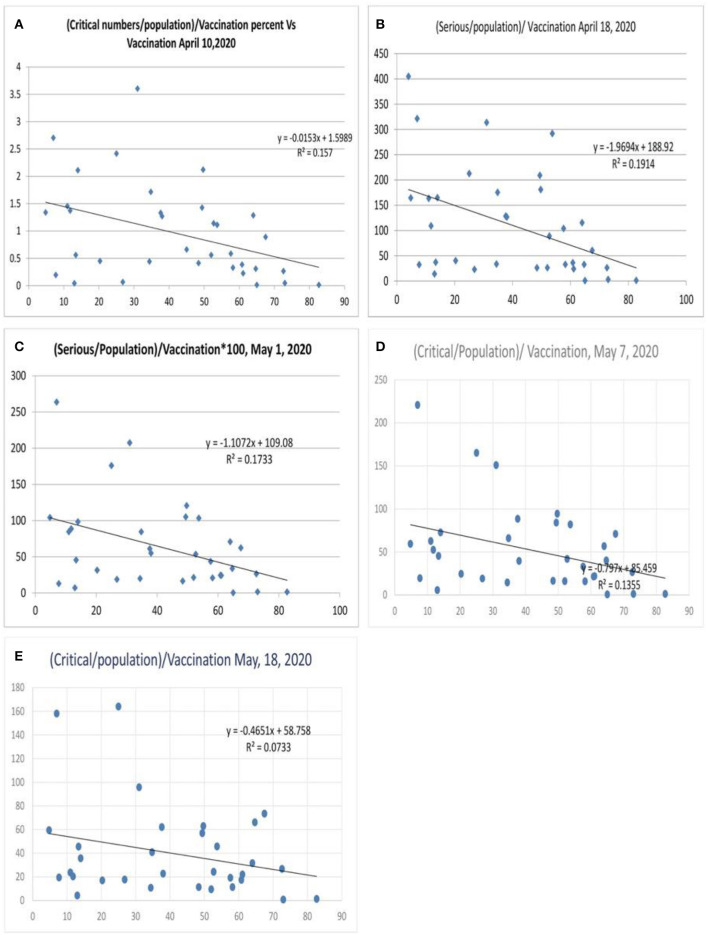
Correlation between influenza vaccination percentage and (critical number/million population)/vaccination percentage [**(A)** April 10, 2020, **(B)** April 18, 2020, **(C)** May 1, 2020, **(D)** May 7, 2020, and **(E)** May 18, 2020].

When morbidity was analyzed (critical numbers/million population, Figures 7A–E), the correlation was lesser (Figure 7A), with a variance of 15.7% benefit by influenza vaccination. A subsequent analysis after 1 week showed an increase in the equation's slope and intercept parameters (Figure 7B) with a variance of 19.1% and further values were 17% and 13.5% (Figures 7C,D) and the value was 7.3% on May 18, 2020 (Figure 7E). There could be reasons for this observation. Mortality is a discrete and finite variable, and the critical/serious position of the variable is a continuous variable subject to change with the progress of the disease, which could be improvement or death. Hence, a better parameter to assess morbidity would be the total number of critical cases so far/population, which is an ideal parameter required for the morbidity correlation. However, this parameter was not available for evaluation, which is a limitation of this study.

**Figure 8:**
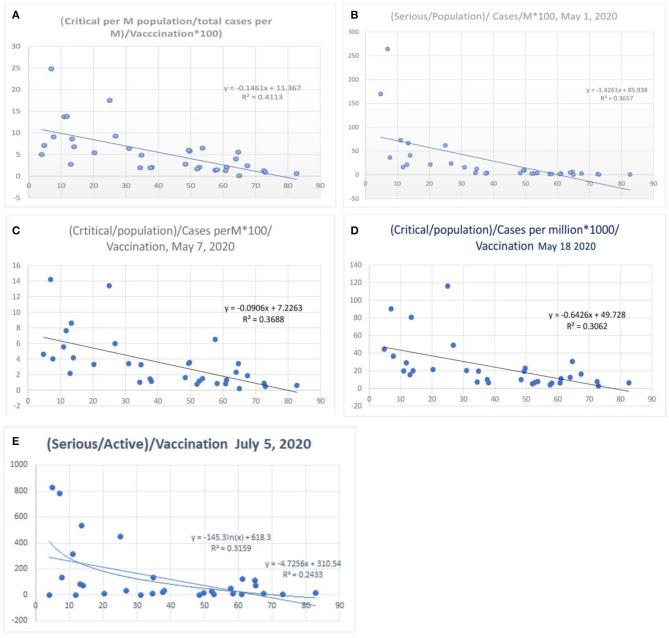
Correlation between influenza vaccination percentage, and [(critical number/million population)/(total cases per million)]/vaccination percentage [**(A)** April 26, 2020, **(B)** May 1, 2020, **(C)** May 8, 2020, **(D)** May 18, 2020 and **(E)** July 5, 2020].

When the severe or critical case numbers were adjusted to the total number of cases per million population (case-morbidity ratio), the correlation was higher with an *R*^2^ of 0.41 (Figure 8A, worldometer data April 26, 2020) and a value fluctuating around 0.34 (*r* =-0.58, Figure 8; May 7, 2020) with the latest value being 0.31 (May 18, 2020). The ratio of critical illness numbers to active case numbers also showed a declining trend with vaccination. Figure 8E shows the *R*^2^ and logarithmic *R*^2^, which are 0.24 and 0.32, respectively (July 5, 2020). This suggests a possibility for higher morbidity reduction. When the morbidity parameters were studied in a gap of 24 h there were minimal changes in the *R*^2^ values (Supplementary Figure S1, 2020) of the morbidity parameters. The exact nature of the critical illness is not reflected in data. However, the primary parameter for morbidity or critical illness is the need for ventilators, oxygen, superadded infections, and multiorgan involvement as well as the presence of uncontrolled associated co-morbidities. Hence, the vaccination strategy can reduce the critical illness parameters.

### COVID-19 Recovery

The recovery data was analyzed, and it was adjusted to the cases per million and tests performed per million. The recovery data of the UK and Netherlands are not available in worldometer data to date (May 10, 2020), and some countries have not updated their recovery data. Also, recovery is a soft parameter that is subjected to over- or under-reporting. With the available data and removal of two extreme variables in data (> 4 or 5 times of the maximum), the graph obtained shows a benefit for vaccination (*R*^2^ 0.06, after adjustment to tests, Supplementary Figure S2, lower panel, May 10, 2020), and *R*^2^ was 0.035 on May 18, 2020.

### Herd Immunity

Antibody testing, when performed in New York, showed about 20% of the population developing antibodies^7^. Similar tests performed in Sweden reveals antibody levels of about 7.3% with highest levels in Stockholm, and maximal antibody levels were seen in the age group 20 to 64 years^8^. In both instances, the tests were performed in a limited number of subjects, i.e., 3,000 and 1,200 subjects, respectively. In Spain, the seroprevalence of IgG antibodies for SARS-CoV-2 was only about 5% in general population, and, among healthcare workers, it was about 11% (24). The neutralizing antibody levels tend to reduce to about 17% after 65 days of infection (25).

Tegnell's method of limited lockdown in Sweden, though it had higher mortality in the initial stages of coronavirus pandemic, could impact T-cell immunity. The benefit could be more when supplementing with the H1N1 vaccine in the age group 20–64 years and protecting the elderly until herd immunity for COVID-19 is imminent. T-cell-mediated immunity for SARS-CoV-2 would increase in asymptomatic or mild COVID-19 patients after COVID-19, and this has not yet been quantified in the general population (26). The exact benefit of this method can be quantified as a comparison only when other countries ease the lockdown measures. The other Scandinavian countries can administer the H1N1 vaccination in the adult population, which can reduce mortality, as most countries aim to reduce lockdown.

### Proteomics

A transcriptomic analysis of the host response showed an overlapping expression of differentially expressed genes (DEGs), genetic ontology (GO) terms, and protein–protein interaction (PPI) networks in response to COVID-19, SARS CoV, MERS CoV, Ebola, and H1N1 infections [(27) and Supplement Reference S1]. From the gene network analysis, we could see the uniquely shared GO terms or DEGs associated with the host response of COVID-19 were H1N1-18, MERS CoV-38, SARS-CoV-20, and 28 for Ebola viruses. Among the unique shared genes in the host response, the overlaps between COVID-19 and other infections were H1N1 (43), MERS-CoV (112), SARS-CoV (30), and 116 for Ebola viruses (27). The PPI network and gene enrichment analysis showed an overlap of genes associated with MMP9, ICAM1, IL-6, CXCL1, TNF, CXCL8, TLR1, IRF 7, VEGF A, and TLR2, which were expressed variably with significant overlap [(27, 28) and Supplement Reference S1]. The T-cell and B-cell response and cytokine secretion pathways also exhibited overlaps between influenza A viruses and SARS-CoV-2 (28). Among the five organisms, only H1N1 has an available vaccine, and the vaccines of other organisms are in the research and development stage. The H1N1 vaccine administration can thus prepare the host gene response to COVID-19 infections. Also, H1N1 vaccine-induced stem antibodies can inhibit the stem of hemagglutinin and the neuraminidase segment, which is an exhibition of pleiotropic effect of the vaccine (29).

### Lower Respiratory Tract Infections (LRI)—*Streptococcus pneumoniae* and Influenza Viruses

The incidence of influenza, viral, and bacterial lower respiratory tract infections (LRIs) is much higher in South and Southeast Asian countries than in Western countries (17, 30). Among the viral lower respiratory tract infections, respiratory syncytial viruses (3.4/1000) and influenza virus infections (5.3/1000 people) are common (17, 30). Among the bacterial infections, *Streptococcus pneumonia* infections (26.7/1000) are the most prevalent LRIs. In the elderly, the incidence of *Streptococcus pneumonia* LRI is 72.8/1000, the influenza virus is 15.8/1000, and the respiratory syncytial virus is 3.4/1000.

The South and Southeast Asian countries have a high incidence of LCIs, 48.8 episodes/1000 population, and 45.9/1000 compared to high-income northern America (38.8/1000) and western Europe (28.7/1000). In the elderly population, the statistical numbers for LRIs are 230/1000 and 181/1000 when compared to high-income North America, including Canada (73.2/1000) and Western Europe (101/1000). Hence, in the elderly population, the dichotomy between these countries/regions is more.

### Comparison of Various Regions With LRIs

For comparison, high-income Asian countries, including South Korea, Brunei, Singapore, and Japan, have LRI death rates (109,683/year) similar to high-income North American countries, including Canada (105,127 deaths/year) and comparable to Western Europe, which comprises of 22 countries, at 138 945 deaths/year. In South Asian countries (India, Pakistan, Nepal, Bangladesh, Bhutan, and Afghanistan), it is 589,653 deaths/year, and, in Southeast Asia, it is 209,873 deaths/year (17). Moreover, these published results are well-documented and organized data from reputed centers, predominantly from the urban and suburban population. In the rural community, the differences would be higher.

Influenza vaccination rates are lower in South Asian countries. Since influenza and LRIs are very common, and they will also have high herd immunity for influenza viruses, the vaccination may not be required. Furthermore, overcrowding increases the spread of lower respiratory tract infections. Since diseases themselves are high, the need for vaccination is less. Also, the mortality rates of COVID-19 could increase in these countries in the next few months. However, the population-adjusted COVID-19 deaths would be significantly lower compared to Western countries, which have higher mortality due to the lower incidence of influenza and other respiratory tract infections.

South Korea, Australia, and New Zealand have emerged successfully so far with lower mortality rates. Analyzing the vaccination statistics of these countries is interesting. South Korea has a vaccination rate of 82–86%. New Zealand and Australia have a vaccination rate of about 65 and 73%, respectively, in age groups >65 years. The overall incidence of lower respiratory tract infections in high-income Asian countries is 45%, and it is 43.8% in Australasia. Among the elderly, the LRI rates are 120.6 episodes/1000 and 165.6/1000 in high-income Asian countries and Australasia, respectively, compared to high income in North America and western Europe, which are 73.2/1000 and 101.3/1000 (15). Hence, these countries (South Korea and Australasia) have higher LRI's and also higher vaccination rates, the combination of which could have helped to achieve the results. Moreover, these countries are known for their efficient testing and confinement methods, and the current results are of the early stages of the pandemic.

The central Latin American countries (including Mexico) document a lesser incidence of LRI deaths (43,191 deaths/year or 111/1000 people) and lower influenza vaccination rates. The pandemic started late in Mexico, and it is reporting an increasing number of cases and mortality. Andean Latin American countries and Tropical Latin American countries like Brazil have higher LRI episodes and lower vaccination rates. There is a gradual fall in the incidence of H1N1 in Peru, especially in Lima (31). Also, they have higher mortality than Asian countries but less so than Western European and high-income North American countries.

In Andean Latin American countries (Peru, Bolivia, and Ecuador), even though the LRI episodes are more frequent, they have a vast land area, and there is significantly less overcrowding; the herd immunity for influenza would thus also be less present. The population density is 26 Persons (P)/km^2^ for Peru and 25 P/km^2^ for Brazil, whereas that of India is 464 P/km^2^, Pakistan is 287 P/km^2^, and the United States is 36 P/km^2^. Central, Tropical, and Andean Latin American countries, based on this analysis, are thus also vulnerable, though less so than Western Europe and high-income North America, and it is advisable that we enhance a vaccination with H1N1 with or without a *Streptococcus pneumonia* vaccine.

### COVID-19 Mortality in Small Regions

Among smaller regions, COVID-19 death rates in Andorra (673/million population), the Isle of Man (282/million), San Marino (1238/million), the Channel Islands (270/million), and Bermuda (145/million), which are located in the regions of Western Europe and the US, are high. Sint Maarten with 350/million (Netherlands) and Montserrat with 200/million (UK) also have higher mortality. These small regions record a disproportionately higher mortality rate compared to other small countries with a comparable population.

## Influenza-Attributable LRI

### High-Income North America and Western Europe

How attributable LRIs are to influenza was studied by the counterfactual method in the Global burden of disease study (GBD). From the published article, it could be observed that Western Europe (137.5/100,000, CI 104–174) and high-income North America (281/100,000 population, CI 197–381) have a lower incidence of influenza-attributable LRIs. Countries like Spain (91, CI 65–120), Italy (63, CI 44–85), France (134, CI 95–182), and the UK (222, CI 158–257) have low influenza LRIs per 100,000 population. In the UK, Northern Ireland (139), Wales (132), and Scotland (163) have lower values compared to England (237) per 100,000.40 Among the Scandinavian countries, the influenza LRI rates per 100,000 were 490 for Norway, 191 for Finland, 137 for Denmark, and 167 for Sweden, 40 and their COVID-19 mortality rates were 47/million for Norway, 106/million for Denmark, 59/million for Finland, and 564/million for Sweden.

### Rest of Europe and Asia

Central Europe (358, 251–488), Eastern Europe (2,399, CI 1717–3205), South Asia (1,063, CI 725–1,479), Southeast Asia (1591, CI 1118–2160), Central Asia (1292, CI 853–1652), North Africa, and the Middle East (775, CI 529–1077) have high influenza LRIs per 100,000 population (32). High-income Asian Pacific countries have 146/100,000 (CI 102–197).

The high-income Asian Pacific group has a lower influenza incidence. The influenza LRI incidence is 145/100,000 (CI 98–193) in Japan and 173.7/100,000 (118–241) in Brunei. South Korea has the highest vaccination among the elderly but has an influenza LRI incidence of 141.6/100,000 (CI 99–192.5). Singapore has an incidence of 290 (CI 204–390). However, the population density is very high: South Korea has 510, Singapore 8,300, Brunei 81, and Japan 347 P/Km^2^, which could catalyze a herd immunity response against influenza. In the high-income Asian Pacific group, Japan and Brunei are vulnerable to COVID-19 mortality due to lower influenza LRI incidence. However, the reported COVID-19 death rate to date is very low (8/million) in these two countries. In Singapore, though the influenza incidence is low (290/100,000), compared to South and Southeast Asian countries, it is less. The COVID-19 mortality rate lower at present, but, as the pandemic evolves, the mortality rate could rise.

### Latin American Countries

Central Latin American countries (443, CI 304–615) and Andean Latin American countries (695, CI 477–961) have higher rates of influenza attributable to lower respiratory tract infections per 100,000. Peru 692.9 (CI 477 to 957), Ecuador 748 (CI 512 to 1037), Bolivia 622 (CI 426 to 863) are associated with comparatively higher influenza rates/100,000 based on GBD data (2017), yet with a higher COVID 19 mortality 394, 299, 180/million respectively (July 17, 2020). The death may selectively happen in areas falling at the lower side of the confidence intervals in these countries, which has to be studied. Also there is a decreasing pattern of influenza in these countries in the last 2 years. Peru and Ecuador have lower population density, as discussed before. Adjacent countries like Brazil and Paraguay differ in mortality rates: Brazil's rate is 208/million, and Paraguay's is 2/million population, and their influenza LRI incidences are 268 (CI 181–378) and 738 (CI 498–1034) per 100,000, respectively.

Taiwan (province of China) had high influenza rates of 976/100,000 (CI 681–1315) and has very little COVID-19 mortality (0.3/million population). Also, Taiwan recorded the highest influenza mortality of 12.2/100,000 cases (32). The influenza-attributable LRIs in Vietnam were high at 3,710/100,000 (CI 2,537–5,141), but so far, no COVID-19 mortality has been reported.

Poland (147, CI 101–200), Brunei (173, CI 118–241), Japan (141, CI 98–193), China (151, CI 104–208), Nicaragua (209, CI 139–296), and, among the African countries, Mozambique (297, CI 201–419) and Ethiopia (329, CI 223–454) have lower influenza LRI incidences per 100,000 population. As the pandemic evolves, these countries may encounter higher degrees of severity of COVID-19 infection. In China, though the infections were successfully controlled in Wuhan in the past, the influenza LRI episodes reported are less 151/100,000, and COVID-19's susceptibility and severity could be high as the pandemic evolves. Of late, there has been a mild spike of cases in the Xinjiang province (Urumqi), which could be an early indicator.

Australia (125, CI 86–71) and New Zealand (193, CI 132–267) are associated with fewer influenza-attributable LRIs per 100,000 population. Though these countries have initial success, cases would surge after lockdown, and there is a need for H1N1 vaccination in the adult and elderly population. North Africa (775, CI 529–1,099) and Sub-Saharan countries (590, 408–510) have high rates of influenza LRIs per 100,000, and these countries also have higher population densities. Since the entire data is obtained from the contra factual strategy with a predictive regression model, the values of influenza attributable to LRIs may not be very accurate.

### Correlation of Influenza-Attributable LRIs and COVID-19 Mortality

**Figure 9:**
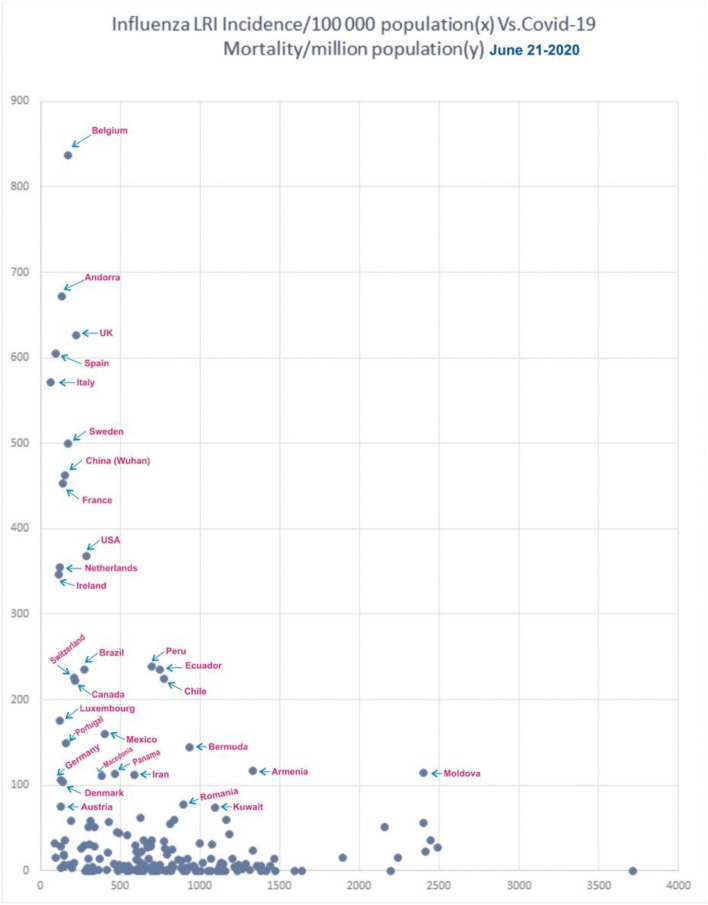
Correlation of influenza incidence/100,000 population and COVID-19 mortality/million population (*n* = 182, June 21, 2020).

Figure 9 shows the incidence of influenza-attributable LRI in population (32) and the COVID-19 mortality. In the Figure 9, for China, only Wuhan population was used. When the influenza incidence is <250 episodes/100,000, a significant rise in COVID-19 mortality is observed. Belgium, Andorra, the UK, Spain, Italy, Sweden, China, France, the USA, the Netherlands, Ireland, Brazil, Canada, Switzerland, Luxembourg, Mexico, Portugal, Panama, Macedonia, Denmark, and Austria (Figure 9) are the countries seeing decreasing levels of COVID-19 mortality.

In the influenza incidence segment of 500–1,000/100,000, the COVID-19 mortality rate was <100/100,000 in most countries. Ecuador, Peru, and Chile were the exception, and they had mortality rates of about 230/million, and Bermuda and Iran showed mortality rates of 145 and 115/million, respectively. Ecuador, Peru, and Chile has population densities of about 25P/Km^2^ each, and, though they had higher influenza rates due to a lower population density, they therefore do not benefit from an active herd immunity against influenza. In countries with higher rates of influenza, such as incidences >1,000/100,000, only Armenia and Moldova were exceptions with a mortality >100/million, and they have a population density of 103 and 123 P/Km^2^, respectively.

### Receiver Operating Characteristic Curve Analysis—Influenza vs. COVID-19 Mortality

**Figure 10:**
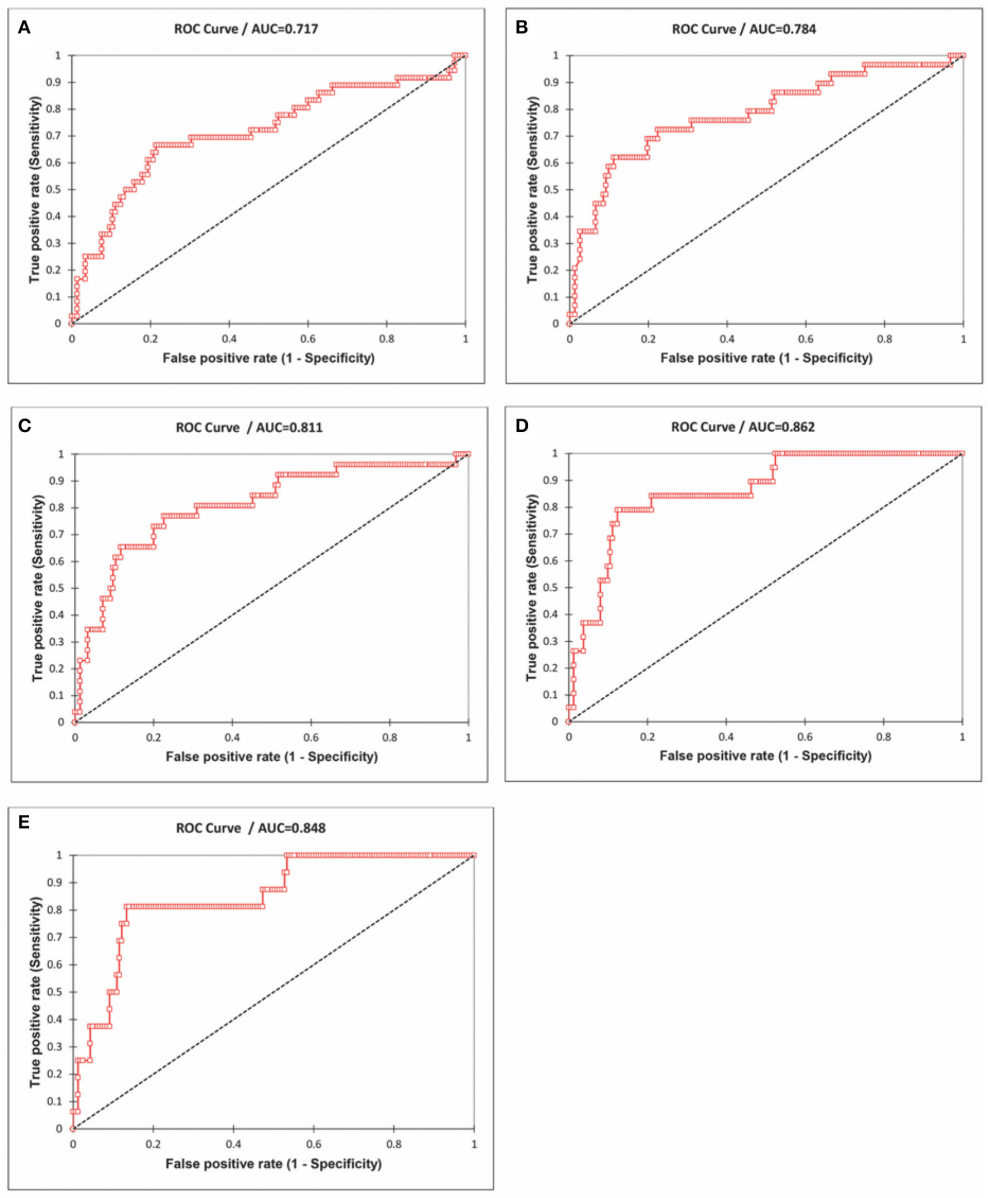
Receiver Operating Characteristics (ROC) curve analysis of the influenza incidence and the mortality rates of COVID-19 (cut-off mortality rate at 50/million, area under the curve-AUC 0.72; **(A)** 75/million, AUC 0.78, **(B)** 100/million, AUC 0.81, **(C)**, 150/million, AUC 0.86, **(D)** 200/million, AUC 0.85, and **(E)** Data June 21, 2020).

The receiver operating characteristic curve (ROC) analysis (Figure 10) showed an increasing trend in the area under the curve (AUC), and, for predicting the mortality >150/million, the AUC was 0.86. The AUC for predicting mortality >200/million is 0.85 for decreasing levels of influenza episodes. At the cut-off value of 290 influenza LRI episodes/100,000, the sensitivity was 79% (CI 0.56–0.92), and the specificity was 88% (CI 0.82–0.92) to indicate COVID-19 mortality. Hence, the lower influenza LRI incidence is associated with higher COVID-19 mortality.

A logistic regression model, including influenza LRI incidence and population density on COVID-19 mortality, was studied. The population density of countries with COVID-19 mortality had no statistically significant impact. To predict COVID-19 mortality >100/million population by influenza incidence/100,000, the odds ratio was −0.78 (CI −1.2 to −0.394, *P* < 0.0001) whereas, for the population density it was −0.08 (CI −0.69 to +0.13, *P* = 0.62); to predict a COVID-19 mortality of >200/million population, the odds ratio for influenza incidence/100,000 was −1.5 (CI −2.42 to −0.81, *P* < 0.0001), whereas, for the population density it was −0.277 (CI −1.65 to +0.128, *P* = 0.59). Though population density does not impact COVID-19 mortality, it can have an impact on individual countries.

### COVID-19 Mortality/Influenza LRIs

**Figure 11:**
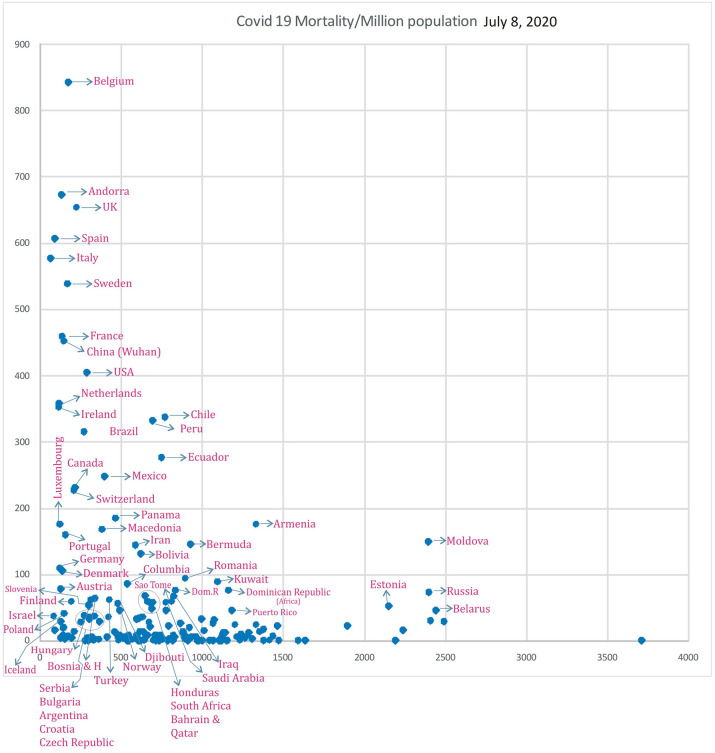
Correlation of Influenza LRI's/100,000 (x) vs. COVID-19 Mortality in various countries (y) (July 8, 2020 *n* = 182).

**Figure 12:**
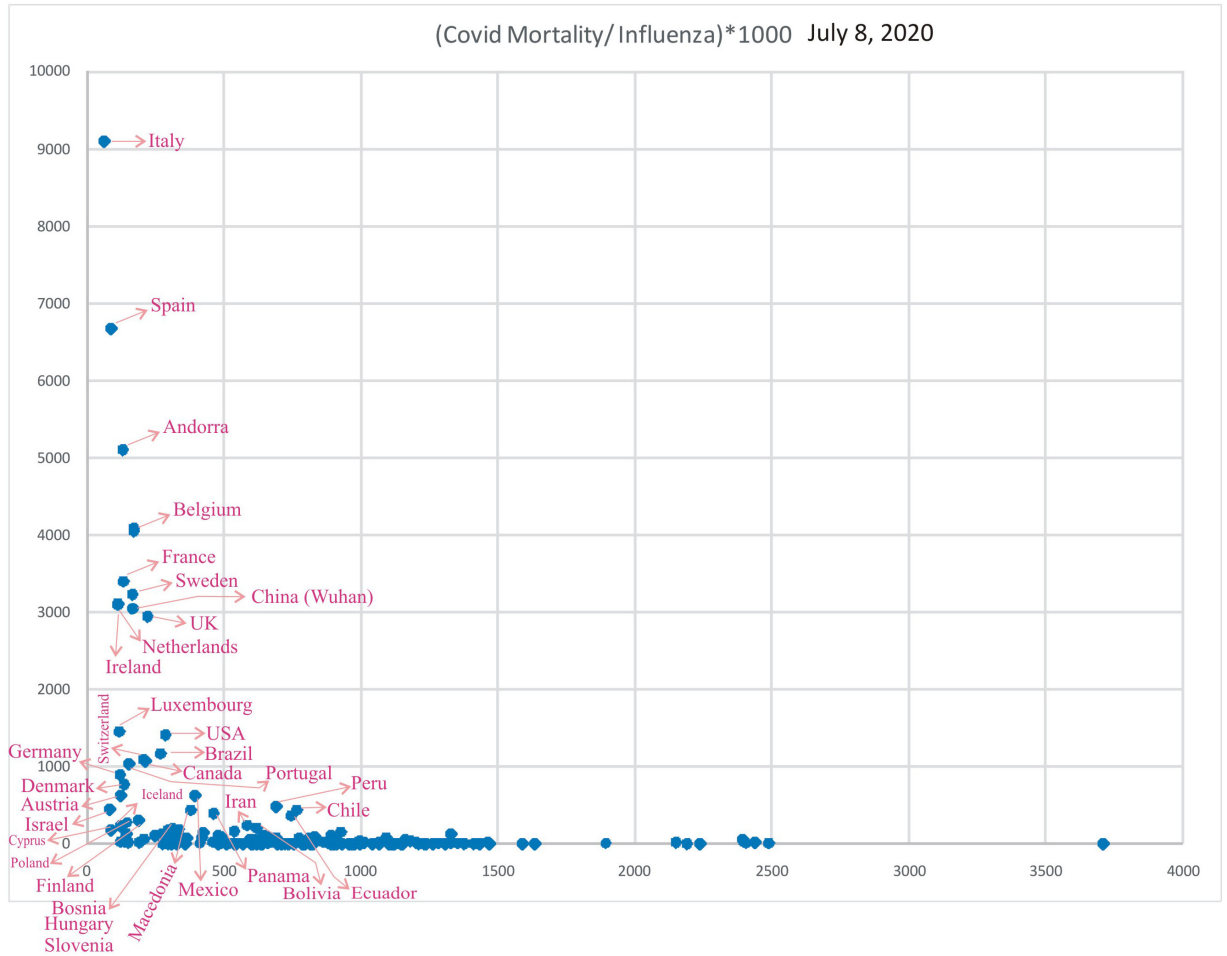
Correlation of Influenza LRI's/100,000 (x) vs. Variable (y), COVID-19 Mortality/Influenza LRI, July 8, 2020 (*n* = 182).

**Figure 13:**
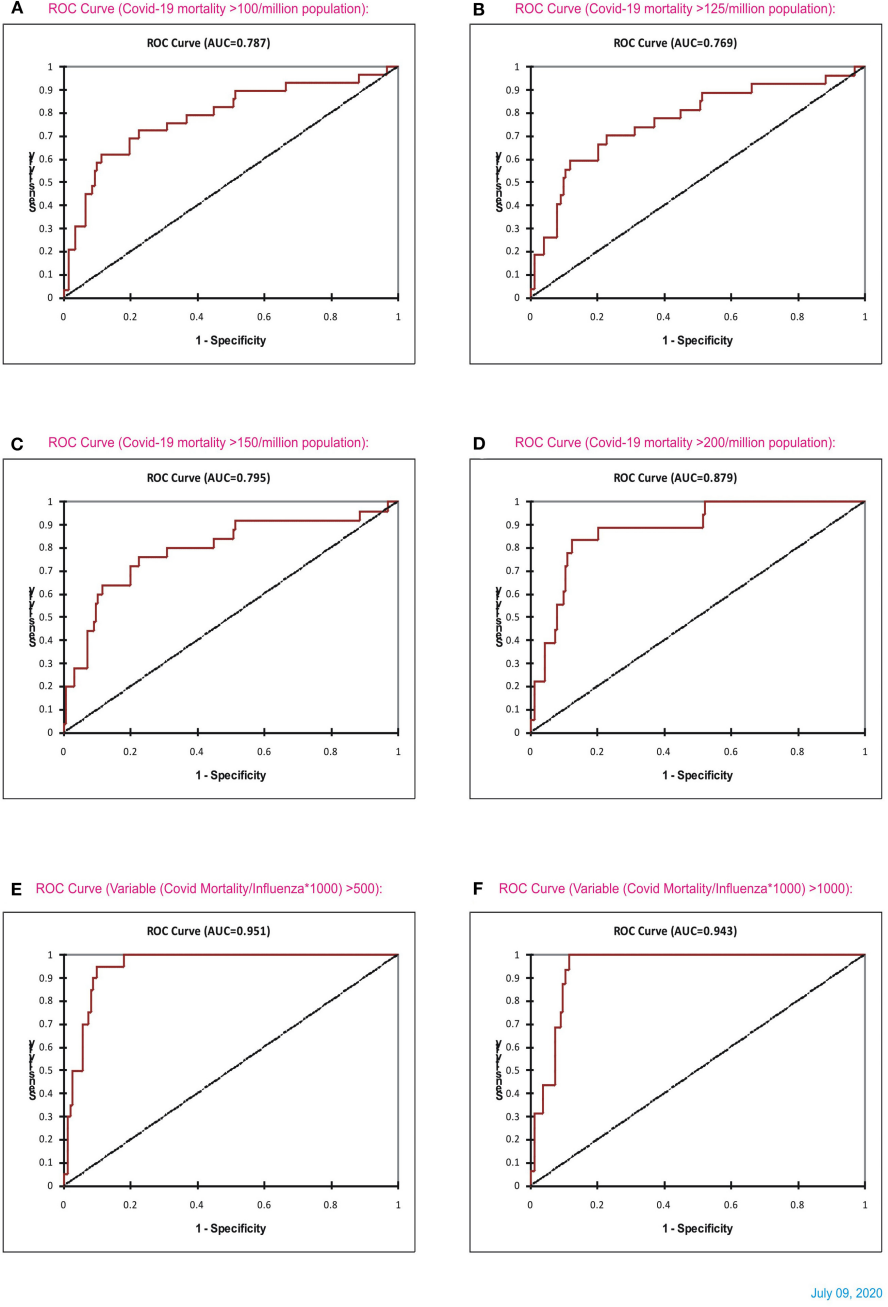
ROC curve statistics of Influenza and COVID-19 mortality **(A–D)**, and ROC curve of influenza LRI vs. parameter (COVID-19 Mortality/Influenza LRI) (July 8, 2020) for (COVID-19 mortality/influenza*1,000) parameter >500 and > 1,000 [**(E,F)**, respectively].

Subsequently, analysis of COVID-19 mortality and influenza LRI episodes continued to show a correlation between the two parameters (Figure 11), which showed a similar trend. When the significance of the influenza parameter is increased using the X (Influenza LRIs) and Y/X (COVID-19 mortality/influenza LRIs) correlation method, there was a higher increasing trend in mortality in countries with fewer influenza episodes (Figure 12). ROC-curve statistics showed a beneficial trend to indicate COVID-19 death by use of the influenza LRIs and COVID-19 mortality of 100, 125, 150, and 200/million population with the area under the curves (AUC) being 0.787, 0.769, 0.795, and 0.879, respectively (Figures 13A–D, *P* < 0.0001, for all, July 9, 2020). When the Influenza LRI episodes were marked to indicate the COVID-19 mortality/influenza LRI episodes parameter >500 and >1,000, the AUCs were 0.951 and 0.943, respectively (Figures 13E,F, *P* < 0.0001, for each). In logistic regression analysis, population density did not correlate (OR −0.33, CI −1.43 to 0.77, *P* = 0.558) with COVID-19 mortality (COVID-19 mortality/influenza LRI episodes >1,000, July 9, 2020), whereas influenza LRI's events were significantly associated (OR −3.8, CI −5.98 to −1.67, *P* = 0.001). For COVID-19 mortality/influenza LRI episodes >500, the Influenza LRI values were OR −3.8, CI −5.7 to −1.9, *P* < 0.0001, and population density OR was −0.45 (CI −1.54 to 0.64, *P* = 0.4). To predict the parameter, the COVID-19 mortality/Influenza LRI episodes^*^1,000 value > 1,000, the influenza LRI parameter had an odds ratio of −3.83 (CI −5.98 to −1.67). To predict COVID-19 mortality >100/million population by influenza incidence/100,000 (July 9, 2020), the odds ratio was −0.79 (CI −1.2 to −0.38, *P* < 0.0001), whereas, for the population density, it was −0.09 (CI −0.42 to +0.24, *P* = 0.59); to predict COVID-19 mortality of >200/million population, the odds ratio for influenza incidence/100,000 was −1.86 (CI −2.75 to −0.96, *P* < 0.0001), whereas, for the population density, it was −0.35 (CI −1.4 to +0.69, *P* = 0.51).

Countries with a COVID-19 mortality rate lower than 300/million with fewer influenza LRI episodes could experience a surge in cases and deaths when they ease their lockdown measures (co-ordinates of countries in Figures 11, 12). They are also susceptible to more mortality if there is a surge of a second wave, which could be imminent. However, in general, influenza vaccination should be useful to all since influenza LRI episodes have a protective effect.

### Burden of *Streptococcus pneumonia* LRIs and COVID-19 Mortality

In the current scenario, the spread of the coronavirus and problems are higher in countries where the *Streptococcus pneumonia* infection rates are low, i.e., <100/DALYs/100,000 (DALY-disability adjusted life years), with only a few countries being an exception. The mortality of COVID-19 is higher in countries with lower rates of respiratory tract infection and lower rates of bacterial and viral infections (<200 DALYs/100,000) (8). Italy, Spain, and some neighboring countries recorded the lowest number of *Streptococcus pneumonia* infections (<10 DALYs/100,000) (8). South American, African, and many Asian countries have a rate of >1,000/DALYs/100,000. The current COVID-19 case-fatality rates (June 26, 2020 worldometer) are 7.98% in Europe, 5.6% in North America, 3.92% in South America, 2.6% in Africa, 2.5% in Asia, and 1.36% in Oceania.

Further latest data (Supplementary Figures S3–9, July 31 and August 1, 2020) are available in the accompanying supplement, which shows a similar pattern of influenza incidence and Covid-19 mortality. Population density did not have any effect on the Covid-19 outcome. However, the product influenza incidence* population density has an impact on Covid-19 mortality, i.e., decreasing product values were associated with higher Covid-19 mortality (Supplementary Figure S3, logistic regression odd's ratio −2.7 CI −4.86 to −0.53, *P* = 0.01, ROC AUC 0.7). The case incidence (Supplementary Figure S4), mortality (Supplementary Figure S5), and case-fatality ratio (Supplementary Figure S6) were showing beneficial patterns as before, and the benefits were reduction by 24%, 9% and 40% (Logarithmic R-square values, July 31, 2020). Supplementary Figure S7 shows a recent rise in Covid-19 mortality, especially in Peru, Chile, Bolivia, Ecuador, Iran etc., in the influenza incidence segment 500 to 700/100000. After that, the influenza parameter's significance was increased in Covid19 mortality/ Influenza Vs. Influenza, i.e., in a Y/X Vs. X fashion; the higher values of the parameter, were seen in locations with less influenza incidence (Supplementary Figure S8). ROC curve to indicate Covid-19 mortality (>200/million) showed similar results with slight truncation (AUC 0.764, *P* < 0.0001 for > 200/million Covid-19 mortality and AUC 0.782, *P* < 0.0001 for > 250/million Covid-19 death) primarily due to rising cases in countries shown in Supplementary Figure S7. Logistic regression showed an odd's ratio of −0.8 (−1.24 to −0.36, *P* < 0.0001) for influenza parameter to indicate Covid-19 mortality and for the parameter Covid-19 mortality/ Influenza >1000, influenza parameter has an odd's ratio of −3.44 (CI −5.52 to 1.35 *P* = 0.001) and for the parameter Covid-19 mortality/ Influenza >500, influenza parameter has an odd's ratio of −2.68 (CI −3.86 to 1.498, *P* = 0.001). In a study, influenza vaccination is effective in reducing Covid-19 mortality in a small cohort of about 30,000 patients with an odd's ratio reduction for Covid-19 death of 17%, the need for ventilators by about 18%, and the need for critical care by about 8% (Supplement Reference S2). Interestingly, in some of these patients, influenza vaccination was also given at the onset of Covid-19 symptoms.

Supplement Figure S9 shows the latest (August 1, 2020) statistics on Covid-19 mortality/influenza incidence, which is similar to the previous observations. In the logistic regression analysis, the influenza LRI parameter was associated with a significant odd's ratio. The population density had a trend to be associated with Covid-19 mortality. However, it was not statistically significant (Supplement Figure S10). Population density * influenza LRI incidence parameter shows a unique pattern wherein lower values were associated with higher Covid19 mortality (Supplement Figure S11). The influenza LRI incidence*population density parameter had an odds ratio of −2.5 (−4.78 to −0.217, *P* = 0.03, August 1, 2020, Supplement Figure S12) to indicate a Covid-19 mortality >250/million. Similar results were seen with logistic regression for the parameters of Covid-19 mortality >250/million, >200/million, and death every <3000 × people, which was performed on August 14, 2020, using the parameters-influenza LRI incidence, population density, and population numbers (Supplement Figure S13). The Supplement Figure S14 (August 14, 2020) shows the logistic regression analysis results for the influenza LRI parameter to indicate Covid19 mortality, i.e., mortality every <2000 and <5000 × people. Population density * influenza LRI parameter had statistical significance (odd's ratio −1 CI −2 to 0, *P* = 0.048) to show mortality every <6000 × people. It also showed a trend toward significance with Covid-19 death cut off in every <5000 and <3000 × people (August 15, 2020). Individually influenza LRI parameter was significantly associated with Covid19 mortality for all cut-off values with a good model fit (Supplement Figure S15). Lower respiratory tract infections' incidence and mortality in various WHO regions (Supplement Figure S16, August 16, 2020) showed a higher incidence of LRIs and LRI related mortality were associated with lower Covid19 mortality. Supplement Table S1 shows the latest Covid19 mortality, influenza vaccination rates, and population in a million.

## Discussion

This study identifies a mild beneficial correlation between influenza vaccination and COVID-19 mortality. The incidences of influenza-attributable LRIs and lower respiratory tract infections, especially of *Streptococcus pneumonia*, are associated with less COVID-19 mortality. In the cohort of US military who receive influenza vaccination regularly (≅100%) and health care professionals with high vaccination rates (about 81%), the COVID-19 mortality is the lowest. The US military cohort is a well-monitored cohort, and it includes mortality associated with various co-morbidities. This is unlike in a similar general population cohort in any country, where death at home and other associated conditions like acute cardiovascular, cerebrovascular events, etc. may not be included or tested. With the highest influenza vaccination rates (82 to 86%, age ≥ 65 years), South Korea has consistently low mortality in the pandemic. The possible mechanism of protection is possibly by modulating the immunity of the individuals, which could be innate or adaptive (11). There is thus a possibility for an influenza (H1N1) vaccine for partial protection against coronaviruses, especially in countries with a low incidence of lower respiratory tract infections or influenza LRIs. In appropriate circumstances, a *Streptococcus pneumonia* vaccine can be also be supplemented (9). Recent study shows a significant declining trend in the antibody levels after primary infection (33). Hence, H1N1 vaccination would be a useful measure to reduce COVID-19 mortality.

High-income countries or countries with a low incidence of LRIs or influenza-attributable LRIs thus depend on influenza vaccination for immunity. Influenza vaccination is therefore recommended in these high-income countries of North America, including Canada, and Western Europe (including the UK and Scandinavia) for the adult population alongside enhancement of immunization for the elderly to reduce COVID-19 severity. Central Latin American and Andean Latin American countries would also benefit from H1N1 immunization. Australia, New Zealand, Brazil, China, Japan, Poland, and Brunei would also to benefit from this immunization strategy. In low-income countries, high-risk and high-income groups could be vaccinated to extrapolate the benefits of these observations since the mortality benefit for COVID-19 is a significant end-point. Therefore, even in low-income countries with a higher incidence of influenza, until a dedicated vaccination for COVID-19 is available, H1N1 immunization would a useful strategy.

### COVID-19 Case-Fatality Rate

In the case fatality (mortality/cases) rate, the influenza LRI parameter was consistently associated with COVID-19 mortality. The denominator of the case fatality rate, i.e., the incidence of COVID-19 instances, is dependent on the number of COVID-19 tests performed. In many of the low-income countries, the COVID-19 tests performed are less compared to the high-income western countries. Hence, if a correction is performed, the case-fatality rate associated with low influenza LRI parameter values would be higher.

### Influenza Burden

The current study focusses on LRIs and influenza-attributable LRIs. The overall burden of influenza could vary, and it is based on the reportage in hospitals and healthcare system, which differs significantly in different countries. Germany experienced influenza-attributable medically attended acute respiratory illness (iMAARI) in the 2018/2019 season in about 3,800,000 (CI 3.0 to 4.6 million) people, and physician-certified influenza-associated incapacities to work were estimated to be 2.3 million (CI 2.1 to 2.5 million)^9^. A sizable number would have minimal symptoms or be asymptomatic. Also, the avian influenza incidence was higher especially in the eastern sides of Germany (Supplement Reference S3). Germany has a population density of 240 P/Km^2^. Influenza-attributed illness is much higher than the calculated GBD data, which is determined as 101,000 LRI episodes in a year (2017) (32). The higher incidence of influenza could be the immunological mechanism of Germany's lower mortality rates despite lower influenza vaccination rates (37%) in the elderly (≥65 years). Israel's influenza LRI incidence by GBD data is 83.2/100,000, and it is susceptible to higher Covid-19 mortality. The current incidence of influenza in Israel by Israel CDC data, including all age groups, is about 65/10 000, or 650/100,000 in 2020. This could possibly be the reason for current low mortality in Israel as well^10^. Nevertheless, in both Germany and Israel, since the GBD data shows a lower incidence of influenza (<150/100,000), there is a susceptibility for higher mortality as the pandemic evolves.

Among Peru, Bolivia and Ecuador, there is a significant reduction of the number and frequency of samples positive for influenza in the last two years, i.e., 2018 to 2020 compared to the previous four years (https://apps.who.int/flumart/Default?reportNo=7). Possibly this reduction of the trend in influenza could reflect higher Covid-19 mortality results. Also, in these three countries, the influenza mortality (2.8, 1.7, and 2.3/100,000 respectively) and hospitalization rates (37, 36, and 30/100,000 respectively) are comparable to or less than those in High-income North America and Western Europe reflecting a reducing influenza burden. New Zealand also has a similar trend with the influenza samples. Australia, New Zealand, Serbia, Poland, Turkey, and Central American countries, also have the same pattern in influenza hospitalisations and mortality data, especially with an influenza incidence of about 500/100,000 or less. As the pandemic evolves further, they are susceptible to higher mortality (32).

The COVID-19 mortality also depends on other parameters like the healthcare system, people's adherence to lockdown measures, and comorbidities. However, COVID-19 mortality rates are higher in countries with well-developed healthcare systems. Hence, the influenza burden could exert a significant contribution to this issue.

Further additional discussion is available in the section of Supplement Discussion.

### Correlation Methods (X and Y vs. X and Y/X Method)

A recent study published (34) observed a positive correlation between influenza vaccination and COVID-19 mortality in Europe (*r* = 0.68 and *R*^2^ = 0.46) and in the US (*r* = 0.29, and *R*^2^ = 0.084). Also, in that study, there was a mild correlation in the case fatality ratio with vaccination in Europe (*r* = 0.38 and *R*^2^ = 0.14). There was no correlation in the case fatality rate in the US (*r* = 0.19 and *R*^2^ = 0.036). The study (34) includes more Eastern European countries with low vaccination rates: Romania, Cyprus, Malta, Bulgaria, Greece, and Poland. The current paper, however, includes statistics of Australia, New Zealand, Canada, Chile, and Iran, and the abovementioned eastern European countries are not included. The approximate population in the >65 years category is 20%, and the vaccination is in a low percentage of these patients only. COVID-19 mortality, however, occurs in the total population irrespective of age. Hence, a direct linear correlation of influenza vaccination and COVID-19 mortality may not be significant. Hence, to increase the significance of vaccination parameter the correlation in this study was performed using the x and y/x method. The current study did observe a negative correlation between vaccination (X) and mortality or case-fatality ratio numbers (Y) in a correlation method of X and Y/X.

### Migrants

The mortality of COVID-19 exponentially increases in the age group >50 years (35)^11^. In the UK and US, the vaccination rates are 72 and 68%, respectively, in the age group of those over 65 years. When the vaccination rates of age group > 50 years are combined, the overall vaccination rates would be about 50% only. Both these countries also have a sizable unregistered migrant population. When this data is connected, the vaccination will fall further. Also, in the high-income countries of Western Europe and the US, there are fewer incidences of lower respiratory tract infection.

### Cost and Strategy

The average cost for H1N1 vaccination is about 30 US dollars. If a *Streptococcus pneumonia* vaccination is included, the total cost of vaccination would be ~60 US dollars or less per person (36). A comparison of this expenditure could be the cost of a few days' worth of face masks used by these individuals. Hence, this vaccination would be a cost-effective strategy used as a preventive measure of immunization in adults and the elderly, including the undocumented migrants, to reduce the global economic burden. A focus on the primary care strategy along with the tertiary care strategy would yield benefits. The regular vaccination schedule for children <5 years need to continue.

### Applications and Limitations

The data collection of COVID-19 mortality varies in different countries, and the accuracy of the data cannot be ensured. However, there is a general pattern of cases and death observed in adjacent countries and regions also, and the data is thus acceptable. Vaccination was performed in age> 65 years, and the COVID-19 mortality was compared with the general population data. The classification of COVID-19 death in the vaccinated or non-vaccinated group is not available. Hence, the vaccination correlation cannot be considered as a conclusive result. Data on influenza vaccination for 2019 was not available. However, a similar trend of immunization has been observed in most countries in the last few years. Nevertheless, the study identifies a mild beneficial correlation with a reduced COVID-19 severity with influenza vaccination and identifies the higher COVID-19 mortality in lower influenza-attributable LRI incidence countries.

The influenza LRI incidence data and the lower respiratory tract infections' burden data is exhaustive and available for most countries (17, 32). This data is thus more robust and thorough evidence for the concept than influenza vaccination data statistics. The population in Europe above 65 years is about 18% (34). The vaccination data covering those aged >65 years may therefore not represent the generalized population since COVID-19 mortality starts increasing after the age of 20.

## Future Perspectives

### Cross-Talk and Chaos

The immune system functions in many axes. Crosstalk between the neutrophils and lymphocytes (37) as well as the microbiome and the immune system is a common phenomenon (38). Also, stimulation of the immune system with these vaccines would efficiently build a defense, crosstalk, and “chaos” (39) in the frontline, which would strengthen the immune system for SARS-Co-2 infections.

CCR5-delta32 polymorphism and expression play an essential role as coreceptor in the virus entry stage of human immunodeficiency viruses (40) and clearance of hepatitis C viruses (41) Short genomic sequences similar to the GP120 of human immunodeficiency virus have been noticed in the genome of SARS-Cov-2 spike proteins (42) CCR5 has a significant role in inflammatory pathogenesis in various systems (43). It is well-known that the SARS-CoV-2 virus entry is through ACE-2 receptors. The role of CCR5 is yet to be determined in SARS-CoV-2 infections. If there is an association between CCR5 and SARS-CoV-2 severity, CCR5 blockers like maraviroc can be studied for its plausible effects. Though CCR5 is a transmembrane protein coupled to a G protein (44) and lacks ubiquitination (45), soluble CCR5 (46) can be studied in plasma as a simpler technique instead of genetic polymorphism during the pandemic.

### G4 (EA) H1N1

The prevalence of Eurasian-like reassortant G4 EA H1N1 swine influenza virus with 2009 pandemic virus genes has been observed recently (47), and there is a potential use for it in human infection. In this scenario, H1N1 vaccines would thus be advantageous.

### Streptococcus Pyogenes

Streptococcus pyogenes have immune regulatory potential; they are also potential candidates for vaccines, and their effect is multifunctional (48, 49). It is worthwhile to investigate the role of these streptococcus pyogenes vaccines against SARS-CoV-2 viruses, which can encompass a delicate balance of nature. The streptococcus pyogenes live vaccines can inhibit the viruses by endonucleases. It could help develop an immune response for host surveillance through M-protein type vaccines (50).

The case numbers are increasing, and, at present, the global incidence of COVID-19 is 23 M. A vast majority of the world's population, however, is not affected. As the lockdown measures are relaxed, the incidence and mortality of COVID-19 very well might increase. SARS-CoV-2 is an evasive candidate for vaccine development, and an RNA virus pandemic was predicted by the scientists already in 2017 (51).

## Conclusion

Currently available influenza and *Streptococcus pneumonia* vaccines have potential to be used as parts of an inexpensive strategy to prevent severe forms of COVID-19 in the general population. These measures could reduce the mortality and morbidity of COVID-19.

## Data Availability Statement

All datasets presented in this study are included in the article/Supplementary Material.

## Author Contributions

MA conceived the idea and method, performed the analyses with statistics, and wrote the paper. The views expressed in the paper are the authors' own.

## Conflict of Interest

The author declares that the research was conducted in the absence of any commercial or financial relationships that could be construed as a potential conflict of interest.

## Footnotes

^1^https://www.satista.com/statistics/1088880/share-of-people-vaccinated-against-influenza-in-belgium

^2^https://www.ecdc.europa.eu/sites/default/files/documents/AER_for_2018_seasonal-influenza-corrected.pdf

^3^https://apps.who.int/iris/bitstream/handle/10665/326242/WER9432-en-fr.pdf?ua=1

^4^CDC.gov. Flu Vaccination Coverage, United States, 2018–19 Influenza Season.

^5^https://www.cdc.gov/flu/professionals/healthcareworkers.htm

^6^https://www.cdc.gov/coronavirus/2019-ncov/cases-updates/cases-in-us.html

^7^https://www.livescience.com/covid-antibody-test-results-new-york-test.html

^8^http://outbreaknewstoday.com/COVID-19-antibody-study-in-sweden-75413/

^9^https://www.rki.de/EN/Content/infections/epidemiology/inf_dis_Germany/influenza/summary_2018-19.htm

^10^https://www.health.gov.il/PublicationsFiles/flu_11012020e.pdf

^11^https://www.worldometers.info/coronavirus/coronavirus-age-sex-demographics

## References

[1] Menachery V Eisfeld A Schäfer A Josset L Sims A Proll S. Pathogenic influenza viruses and coronaviruses utilize similar and contrasting approaches to control interferon-stimulated gene responses. mBio. (2014) 5:e01174–14. 10.1128/mBio.01174-14 24846384 PMC4030454

[2] Zeng Q Langereis M van Vliet A Huizinga E de Groot R. Structure of coronavirus hemagglutinin-esterase offers insight into corona and influenza virus evolution. Proc Natl Acad Sci USA. (2008) 105:9065–9. 10.1073/pnas.0800502105 18550812 PMC2449365

[3] Li F. Structure, function, and evolution of coronavirus spike proteins. Annu Rev Virol. (2016) 3:237–61. 10.1146/annurev-virology-110615-042301 27578435 PMC5457962

[4] Yang P Gu H Zhao Z Wang W Cao B Lai C. Angiotensin-converting enzyme 2 (ACE2) mediates influenza H7N9 virus-induced acute lung injury. Sci Rep. (2014) 4:7027. 10.1038/srep07027 25391767 PMC4229671

[5] Dou D Revol R Östbye H Wang H Daniels R. Influenza A virus cell entry, replication, virion assembly and movement. Front Immunol. (2018) 9:1581. 10.3389/fimmu.2018.01581 30079062 PMC6062596

[6] Ramos I Fernandez-Sesma A. Cell receptors for influenza A viruses and the innate immune response. Front Microbiol. (2012) 3:117. 10.3389/fmicb.2012.00117 22536196 PMC3332393

[7] Liu X Yang N Tang J Liu S Duan Q Wang X. Downregulation of angiotensin-converting enzyme 2 by the neuraminidase protein of influenza A (H1N1) virus. Virus Res. (2014) 185:64–71. 10.1016/j.virusres.2014.03.010 24662240 PMC7114376

[8] Brooks L Mias G. *Streptococcus pneumoniae*'s virulence and host immunity: aging, diagnostics, and prevention. Front Immunol. (2018) 9:1366. 10.3389/fimmu.2018.01366 29988379 PMC6023974

[9] Winje BA Berild J Vestrheim DF Dension E Lepp T Roth A Efficacy and effectiveness of pneumococcal vaccination in elderly – an update of the literature. Norwegian Institute of Public Health. (2019). Available online at: www.fhi.no/en/publ/

[10] Morris DE Cleary DW Clarke SC. Secondary bacterial infections associated with influenza pandemics. Front Microbiol. (2017) 8:1041. 10.3389/fmicb.2017.01041 28690590 PMC5481322

[11] Arokiaraj MC Considering Interim Interventions to Control COVID-19 Associated Morbidity and Mortality – The Perspectives. Available online at: SSRN: https://ssrn.com/abstract=3562102 (accessed March 27, 2020). 10.3389/fpubh.2020.00444 PMC7537040 33072682

[12] OECD Influenza Vaccination Rates (indicator). (2020). Available online at: https://www.cdc.gov/flu/fluvaxview/coverage-1819estimates.htm (accessed July 26, 2020).

[13] Lisewski AM Association Between Influenza Vaccination Rates and SARS-CoV-2 Outbreak Infection Rates in OECD Countries. Available online at SSRN: https://ssrn.com/abstract=3558270 (accessed March 20, 2020).

[14] Aliyari R Imani S Rezaiy T Shariati M Hanifehzad Masooleh P Mirrezaie SM al. Seasonal influenza vaccination uptake and its socioeconomic determinants in patients and staff of hospitals in Shahroud, Northeast of Iran. Shiraz E-Med J. (2019) 20:e82898 10.5812/semj.82898

[15] Zürcher K Zwahlen M Berlin C Egger M Fenner L. Trends in influenza vaccination uptake in Switzerland: Swiss health survey 2007 and 2012. Swiss Medical Wkly. (2019) 149:w14705. 10.4414/smw.2019.14705 30673116

[16] Kunze U Böhm G Prager B Groman E. Influenza vaccination in Austria: persistent resistance and ignorance to influenza prevention and control. Central Eur J Public Health. (2019) 27:127–30. 10.21101/cejph.a5010 31241287

[17] Troeger C Blacker B Khalil I Rao P Cao J Zimsen S. Estimates of the global, regional, and national morbidity, mortality, and aetiologies of lower respiratory infections in 195 countries, 1990–2016: a systematic analysis for the global burden of disease study (2016). Lancet Infect Dis. (2018) 18:1191–210. 10.1016/S1473-3099(18)30310-4 30243584 PMC6202443

[18] Byeon KH Kim J Choi B Choi BY. The coverage rates for influenza vaccination and related factors in Korean adults aged 50 and older with chronic disease: based on (2016). Community health survey data. Epidemiol Health. (2018) 40:e2018034. 10.4178/epih.e2018034 30056640 PMC6232656

[19] Grabenstein J. Immunization to protect the US armed forces: heritage, current practice, and prospects. Epidemiol Rev. (2006) 28:3–26. 10.1093/epirev/mxj003 16763072

[20] Straits-Tröster K Kahwati L Kinsinger L Orelien J Burdick M Yevich S. Racial/Ethnic differences in influenza vaccination in the veterans affairs healthcare system. Am J Prev Med. (2006) 31:375–82. 10.1016/j.amepre.2006.07.018 17046408

[21] Lone N Simpson C Kavanagh K Robertson C McMenamin J Ritchie L. Seasonal influenza vaccine effectiveness in the community (SIVE): protocol for a cohort study exploiting a unique national linked data set. BMJ Open. (2020) 2:e001019. 10.1136/bmjopen-2012-001019 22422920 PMC3307124

[22] Russell T Hellewell J Jarvis C van Zandvoort K Abbott S Ratnayake R. Estimating the infection and case fatality ratio for coronavirus disease (COVID-19) using age-adjusted data from the outbreak on the diamond princess cruise ship, February 2020. Eurosurveillance. (2020) 25:2000256. 10.2807/1560-7917.ES.2020.25.12.2000256 32234121 PMC7118348

[23] Cruise Ships. Available online at: https://en.wikipedia.org/wiki/2020_coronavirus_pandemic_on_cruise_ships

[24] Pollán M Pérez-Gómez B Pastor-Barriuso R Oteo J Hernán M Pérez-Olmeda M. Prevalence of SARS-CoV-2 in Spain (ENE-COVID): a nationwide, population-based seroepidemiological study. Lancet. (2020) 396:535–44. 10.1016/S0140-6736(20)31483-5 32645347 PMC7336131

[25] Sekine T Perez-Potti A Rivera-Ballesteros O Strålin K Gorin J Olsson A Robust T cell immunity in convalescent individuals with asymptomatic or mild COVID-19. bioRxiv. (2020) 10.1101/2020.06.29.174888 PMC7427556 32979941

[26] Seow J Graham C Merrick B Acors S Steel K Hemmings O Longitudinal evaluation and decline of antibody responses in SARS-CoV-2 infection. medRxiv. (2020) 10.1101/2020.07.09.20148429

[27] Alsamman AM Zayed H The transcriptomic profiling of COVID-19 compared to SARS, MERS, Ebola, and H1N1. bioRxiv. (2020). 10.1101/2020.05.06.080960 PMC7728291 33301474

[28] Blanco-Melo D Nilsson-Payant B Liu W Uhl S Hoagland D Møller R. Imbalanced host response to SARS-CoV-2 drives development of COVID-19. Cell. (2020) 181:1036–45.e9. 10.1016/j.cell.2020.04.026 32416070 PMC7227586

[29] Kosik I Angeletti D Gibbs JS Angel M Takeda K Kosikova M. Neuraminidase inhibition contributes to influenza A virus neutralization by anti-hemagglutinin stem antibodies. J Exp Med. (2019) 216:304–16. 10.1084/jem.20181624 30683737 PMC6363425

[30] Krishnan A Kumar R Broor S Gopal G Saha S Amarchand R. Epidemiology of viral acute lower respiratory infections in a community-based cohort of rural north Indian children. J Glob Health. (2019) 9:010433. 10.7189/jogh.09.010433 31131104 PMC6513504

[31] Tinoco YO Azziz-Baumgartner E Uyeki TM Razuri HR Kasper MR Romero C. Burden of influenza in 4 ecologically distinct regions of Peru: household active surveillance of a community cohort, 2009-2015. Clin Infect Dis. (2017) 65:1532–41. 10.1093/cid/cix565 29020267 PMC5850002

[32] Troeger C Blacker B Khalil I Zimsen S Albertson S Abate D. Mortality, morbidity, and hospitalisations due to influenza lower respiratory tract infections, 2017: an analysis for the global burden of disease study 2017. Lancet Respir Med. (2019) 7:69–89. 10.1016/S2213-2600(18)30496-X 30553848 PMC6302221

[33] Long Q Tang X Shi Q Li Q Deng H Yuan J. Clinical and immunological assessment of asymptomatic SARS-CoV-2 infections. Nat Med. (2020). 10.1038/s41591-020-0965-6 32555424

[34] Consortium, EBMPHET, COVID-19 Severity in Europe and the USA: Could the Seasonal Influenza Vaccination Play a Role? Available online at: https://ssrn.com/abstract=3621446 (accessed June 7, 2020).

[35] Guilmoto C COVID-19 death rates by age and sex and the resulting mortality vulnerability of countries and regions in the world. medRxiv. (2020). 10.1101/2020.05.17.20097410

[36] Yue M Dickens B Yoong J Cheng I Chen M Teerawattananon Y Cook A. Cost-effectiveness analysis for influenza vaccination coverage and timing in tropical and subtropical climate settings: a modelling study. Value Health. (2019) 22:1345–54. 10.1016/j.jval.2019.07.001 31806190

[37] Costa S Bevilacqua D Cassatella M Scapini P. Recent advances on the crosstalk between neutrophils and B or T lymphocytes. Immunology. (2018) 156:23–32. 10.1111/imm.13005 30259972 PMC6283649

[38] Zeevi D Korem T Segal E. Talking about cross-talk: the immune system and the microbiome. Genome Biol. (2016) 17:50. 10.1186/s13059-016-0921-4 26987366 PMC4794930

[39] Heltberg ML Krishna S Jensen MH. On chaotic dynamics in transcription factors and the associated effects in differential gene regulation. Nat Commun. (2019) 10:71. 10.1038/s41467-018-07932-1 30622249 PMC6325146

[40] Van Der Ryst E. Maraviroc - A CCR5 antagonist for the treatment of HIV-1 infection. Front Immunol. (2015) 6:277. 10.3389/fimmu.2015.00277 26097475 PMC4456946

[41] Goulding C McManus R Murphy A MacDonald G Barrett S Crowe J. The CCR5-delta32 mutation: impact on disease outcome in individuals with hepatitis C infection from a single source. Gut. (2005) 54:1157–61. 10.1136/gut.2004.055699 15863470 PMC1774905

[42] Pradhan P Pandey A Mishra A Gupta P Tripathi P Menon M Uncanny similarity of unique inserts in the 2019-nCoV spike protein to HIV-1 gp120 and Gag. bioRxiv. (2020). 10.1101/2020.01.30.927871

[43] Vangelista L Vento S. The expanding therapeutic perspective of CCR5 blockade. Front Immunol. (2018) 8:1981. 10.3389/fimmu.2017.01981 29375583 PMC5770570

[44] CCR5-Chemokine (C-C mitif) receptor 5 (gene/pseudogene) Genetics Home Reference.

[45] Petti LM Marlatt SA Luo Y Scheideman EH Shelar A DiMaio D. Regulation of C-C chemokine receptor 5 (CCR5) stability by Lys^197^ and by transmembrane protein aptamers that target it for lysosomal degradation. J Biol Chem. (2018) 293:8787–801. 10.1074/jbc.RA117.001067 29678881 PMC5995508

[46] Tsimanis A Kalinkovich A Bentwich Z. Soluble chemokine CCR5 receptor is present in human plasma. Immunol Lett. (2005) 96:55–61. 10.1016/j.imlet.2004.07.014 15585308

[47] Sun H Xiao Y Liu J Wang D Li F Wang C. Prevalent Eurasian avian-like H1N1 swine influenza virus with 2009 pandemic viral genes facilitating human infection. Proc Natl Acad Sci USA. (2020) 117:17204–10. 10.1073/pnas.1921186117 32601207 PMC7382246

[48] Arokiaraj MC Wilson J A novel method of immunomodulation of endothelial cells using streptococcus pyogenes and its lysate. biorxiv. (2020) 10.1101/2020.05.13.082180

[49] Dale JB Batzloff MR Cleary PP Courtney HS Good MF Grandi G Current approaches to group a streptococcal vaccine development. In: Ferretti JJ Stevens DL Fischetti VA editors. Streptococcus Pyogenes: Basic Biology to Clinical Manifestations. Oklahoma City, OK: University of Oklahoma Health Sciences Center (2016). Available online at: https://www.ncbi.nlm.nih.gov/books/NBK333413 (accessed February 10, 2016).

[50] Vekemans J Gouvea-Reis F Kim J Excler J Smeesters P O'Brien K. The path to group A streptococcus vaccines: world health organization research and development technology roadmap and preferred product characteristics. Clin Infect Dis. (2019) 69:877–83. 10.1093/cid/ciy1143 30624673 PMC6695511

[51] Carrasco-Hernandez R Jácome R López Vidal Y Ponce de León S. Are RNA viruses candidate agents for the next global pandemic? A review. ILAR J. (2017) 58:343–58. 10.1093/ilar/ilx026 28985316 PMC7108571

