# Supplementary material for: Considering Interim Interventions to Control COVID-19 Associated Morbidity and Mortality—Perspectives
Source: Front Public Health. 2020 Sep 22;8:444. doi: 10.3389/fpubh.2020.00444 (PMC7537040; doi:10.3389/fpubh.2020.00444)
Supplement: Supplementary file 1 [file Data_Sheet_1.pdf]

Considering Interim Interventions to Control Covid-19 Associated Morbidity and Mortality- the Perspectives

Mark Christopher Arokiaraj

Supplement Figure 1. Correlation between influenza vaccination percentage and critical numbers evaluated in a 24hour period difference after May 7 on May 8, 2020.

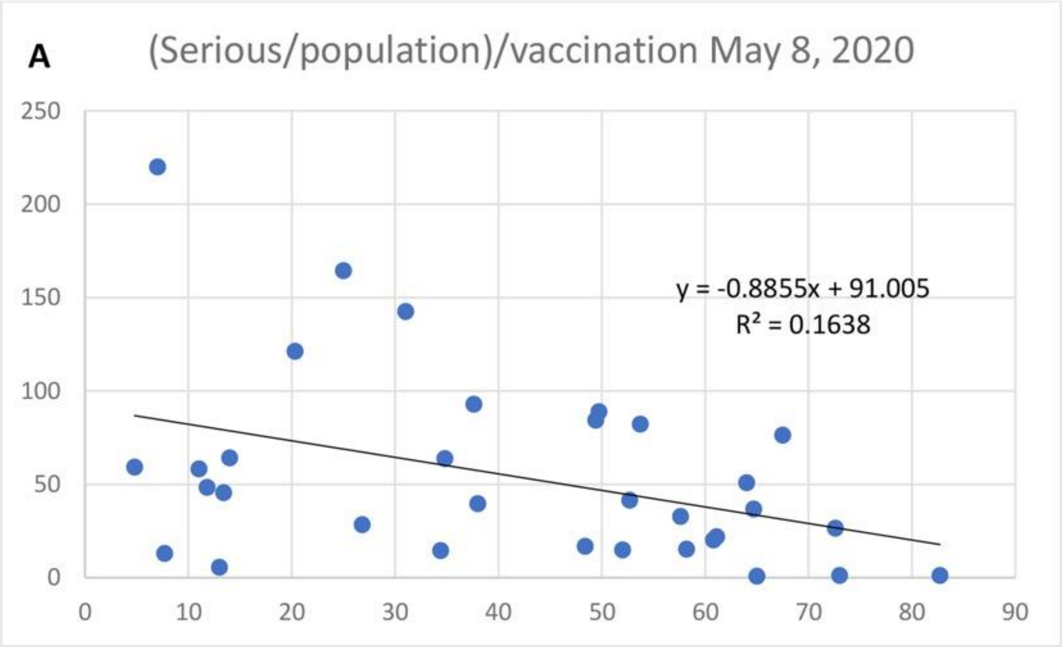

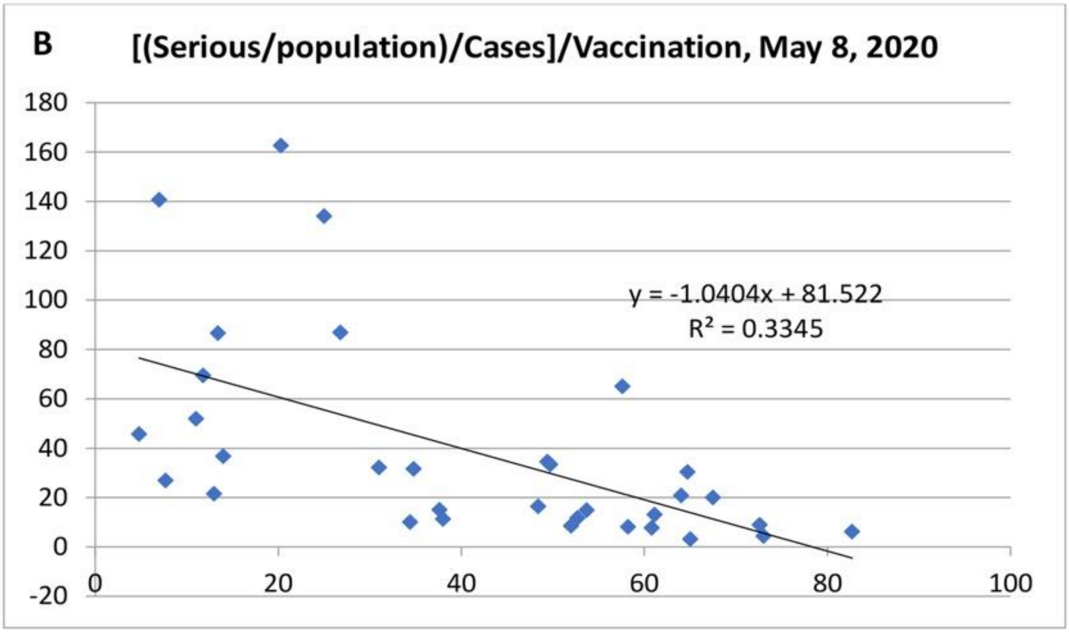

Supplement Figure 2. Correlation between influenza vaccination percentage and recovery parameters and adjusted to tests performed (Panels A and B, May 10, 2020; Panels C and D, May 18, 2020).

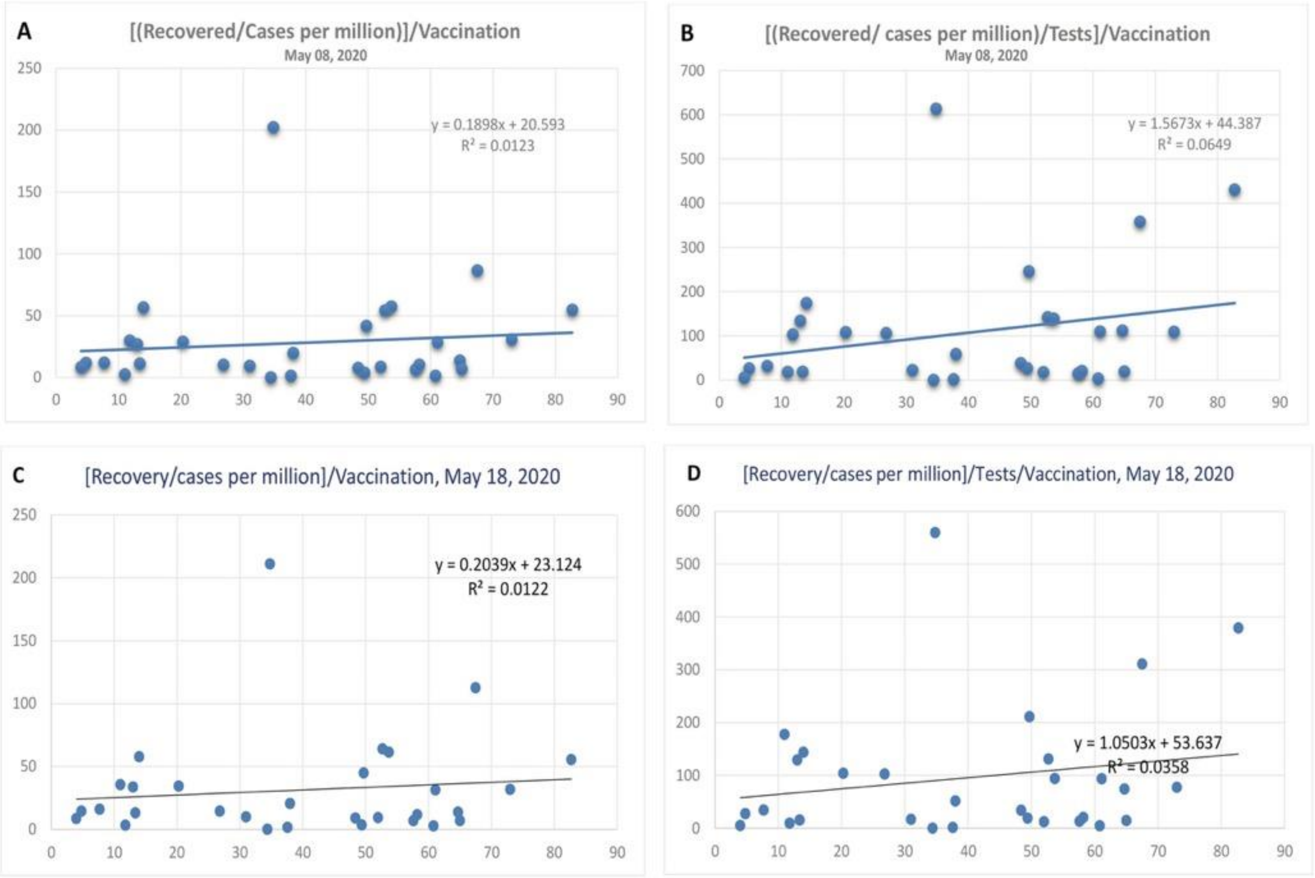

Supplementary figure S3: Influenza lower respiratory tract infections (LRI) incidence/100 000*population density Vs. Covid-19 mortality, lower panel shows the results of logistic regression analysis of this parameter (Influenza LRI incidence/100 000*population density) on Covid-19 mortality 200/million odds ratio -2.7 (CI -4.86 to -0.53, August 1, 2020).

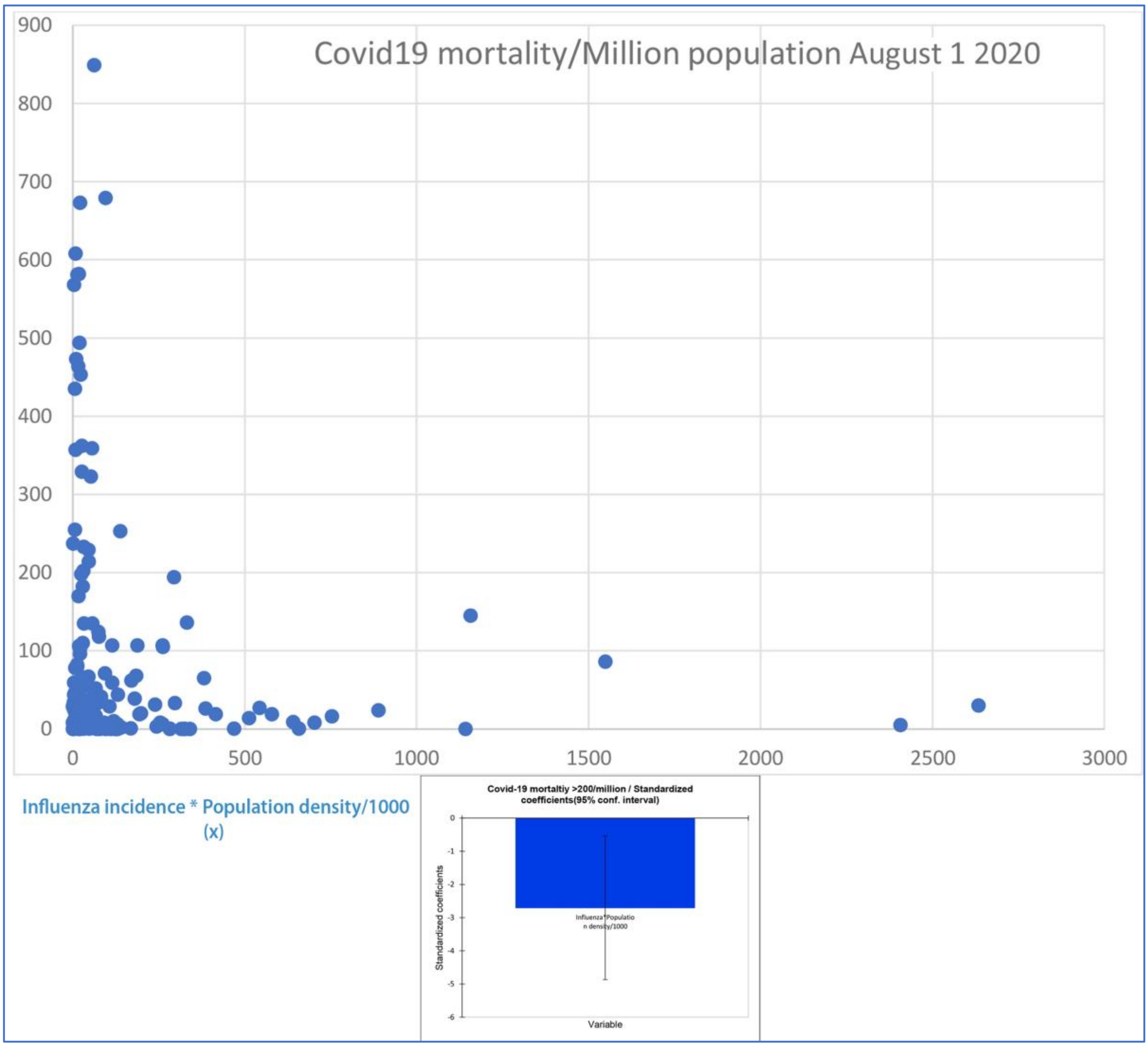

Supplement figure S4: Supplement to figure 1 of the main article.

Influenza vaccination in adults ≥ 65 years Vs. Covid-19 cases per million/vaccination (July 31, 2020).

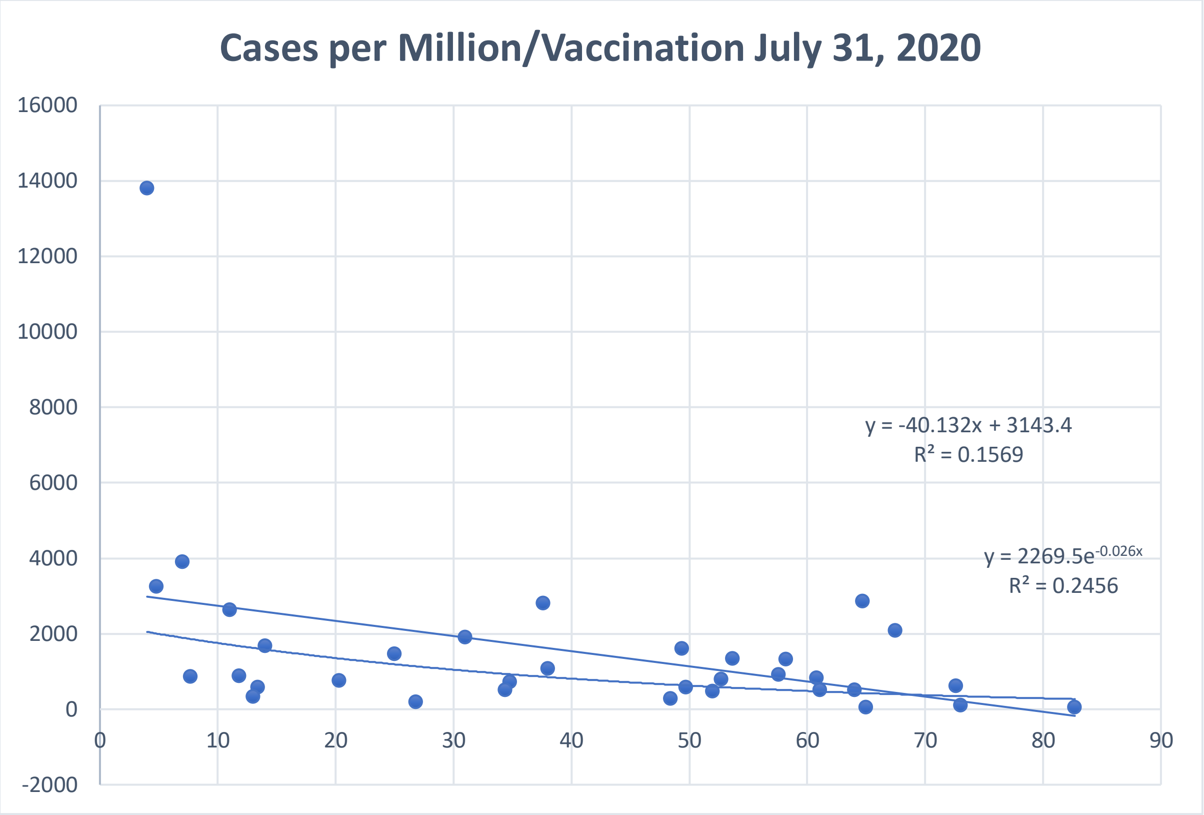

Supplement figure S5: Supplement to figure 2 of the main article.

Influenza vaccination in adults ≥ 65 years Vs. Covid-19 mortality/vaccination (July 31, 2020 and August 13, 2020).

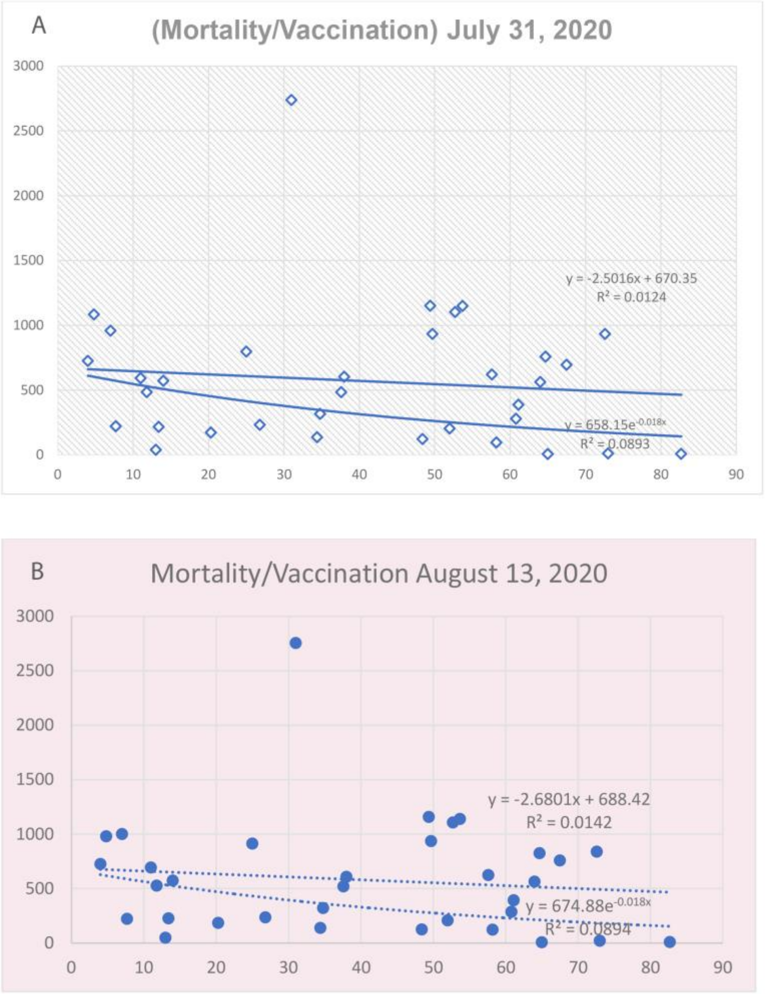

Supplement figure S6: Supplement to figure 3 of the main article. Influenza vaccination in adults ≥ 65 years Vs. (Covid-19 mortality/ Cases)/ vaccination. Linear and logarithmic trend lines are shown (July 31, 2020 and August 13, 2020).

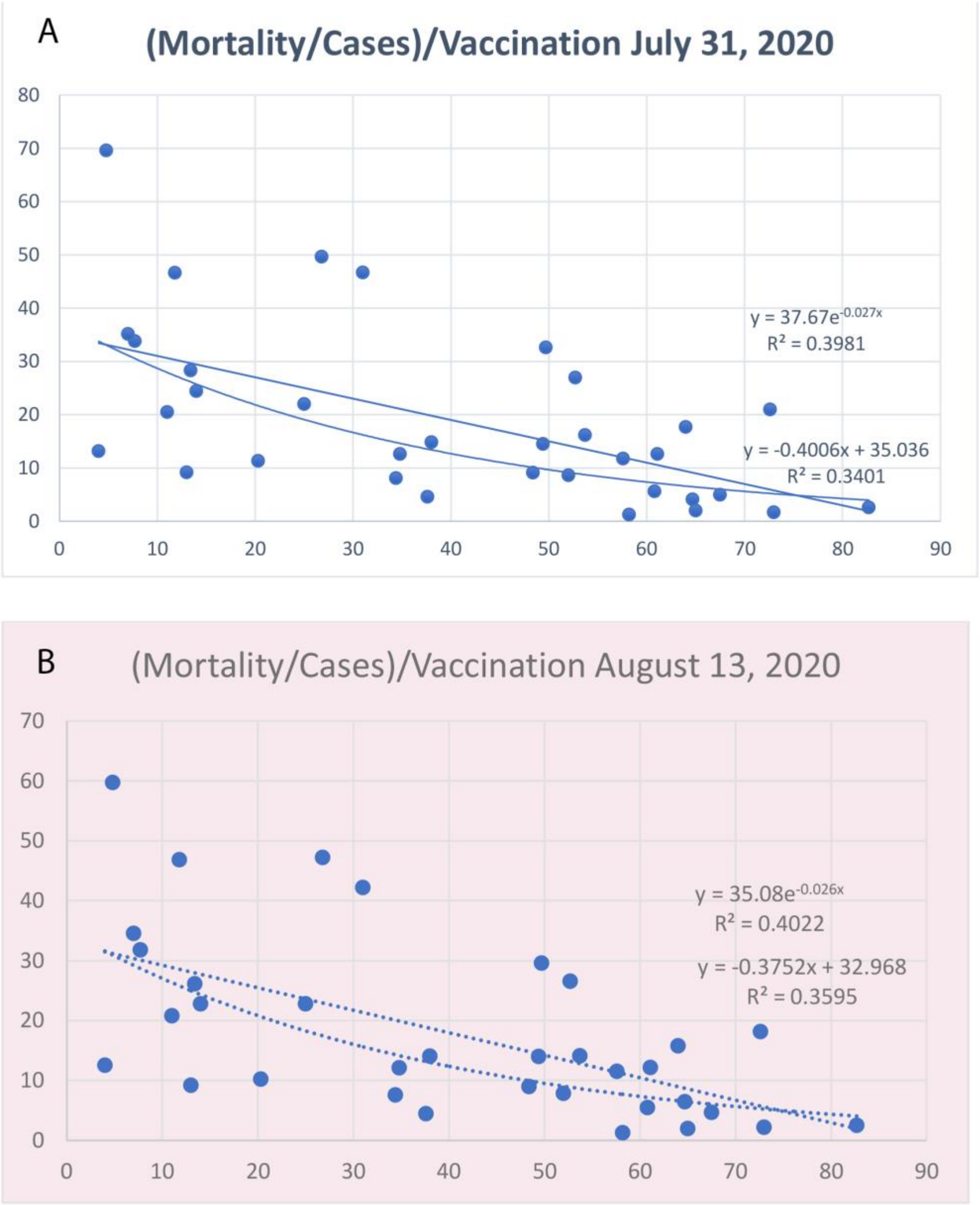

Supplement figure S7: Supplement to figure 11 of the main article.

Influenza LRI incidence and Covid-19 mortality in various countries (N=182), August 1, 2020.

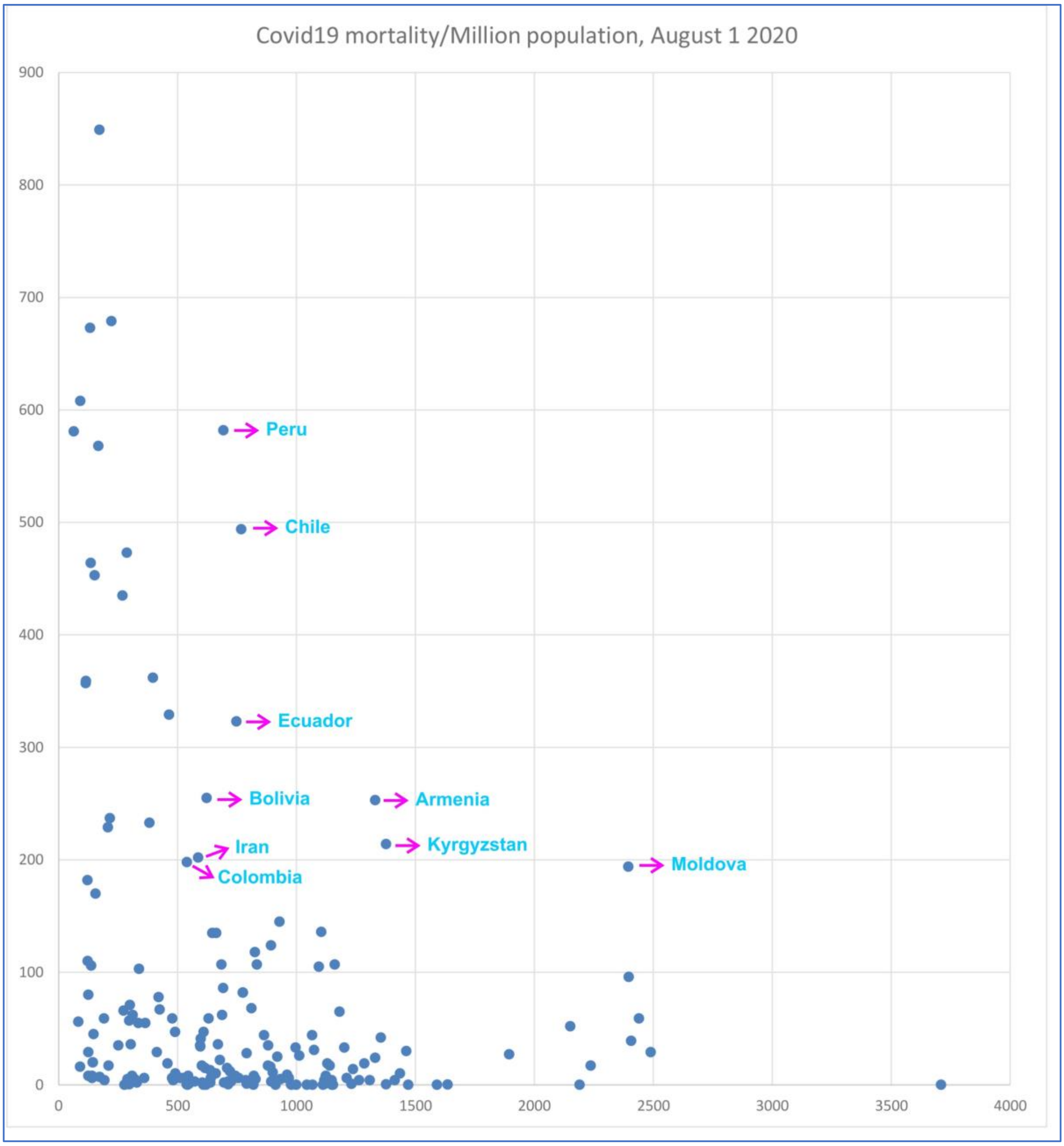

Supplement figure S8: Supplement to figure 12 of the main article.

Influenza LRI incidence Vs. Covid-19 mortality/ influenza LRI incidence(N=182), August 1, 2020.

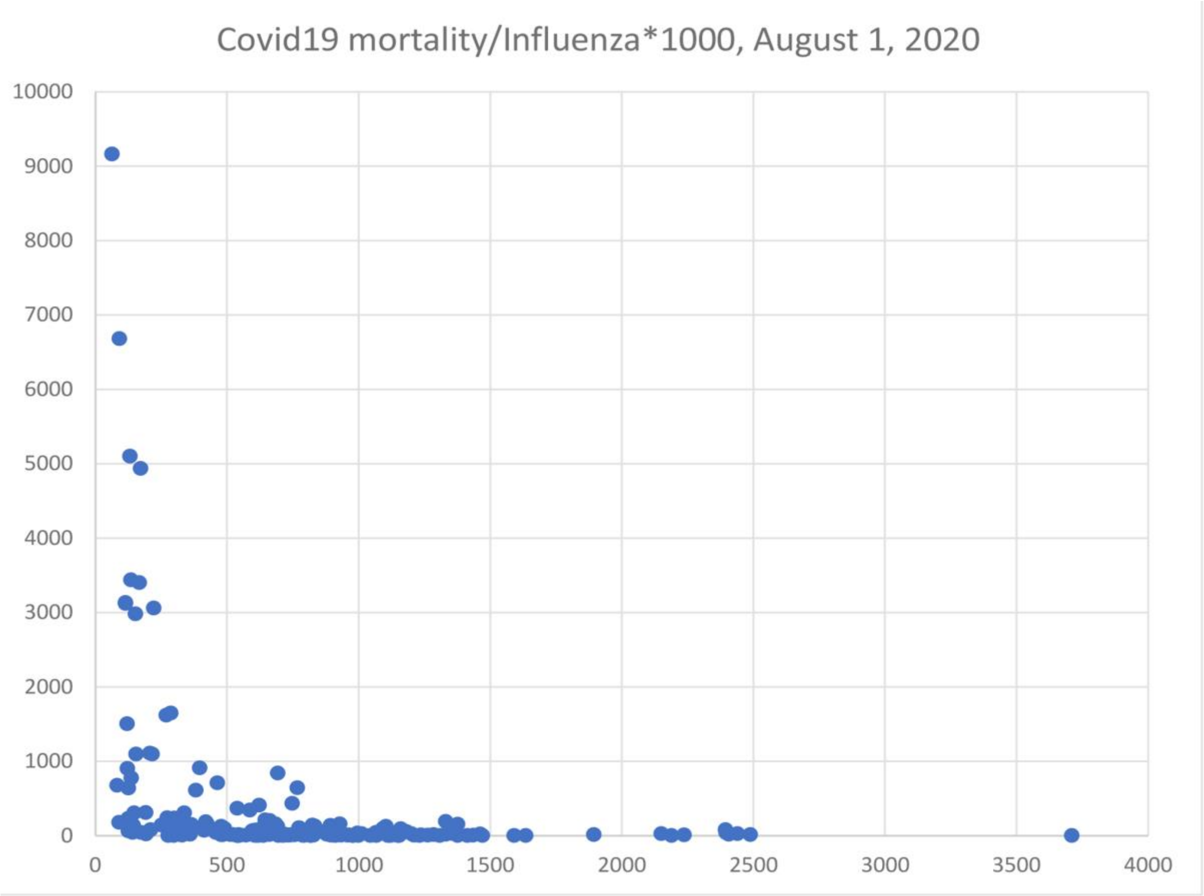

Supplement figure S9: Supplement to figure 13 of the main article.

ROC curve to indicate the Covid-19 mortality(>200/million) with influenza parameter (August 1 2020). Panel A ROC curve, Covid-19 mortality/influenza*1000 >500 (P<0.0001), Panel B - Covid-19 mortality/influenza*1000>1000 (P<0.0001), Panel C - Population density Vs Covid-19 mortality (P=0.29) and logistic regression -0.5 (CI -1.47 to +0.44, P=0.29), Panel D - Covid-19 mortality >200/million Vs Influenza parameter (AUC 0.764, P<0.0001), Panel E - Logistic regression Influenza Vs Covid-19 mortality> 200/million; Odds ratio -0.8 (CI -1.24 to -0.36, P <0.0001, August 1, 2020), Panel F - Influenza Vs Covid-19 mortality>250/million (AUC O.782, P<0.0001).

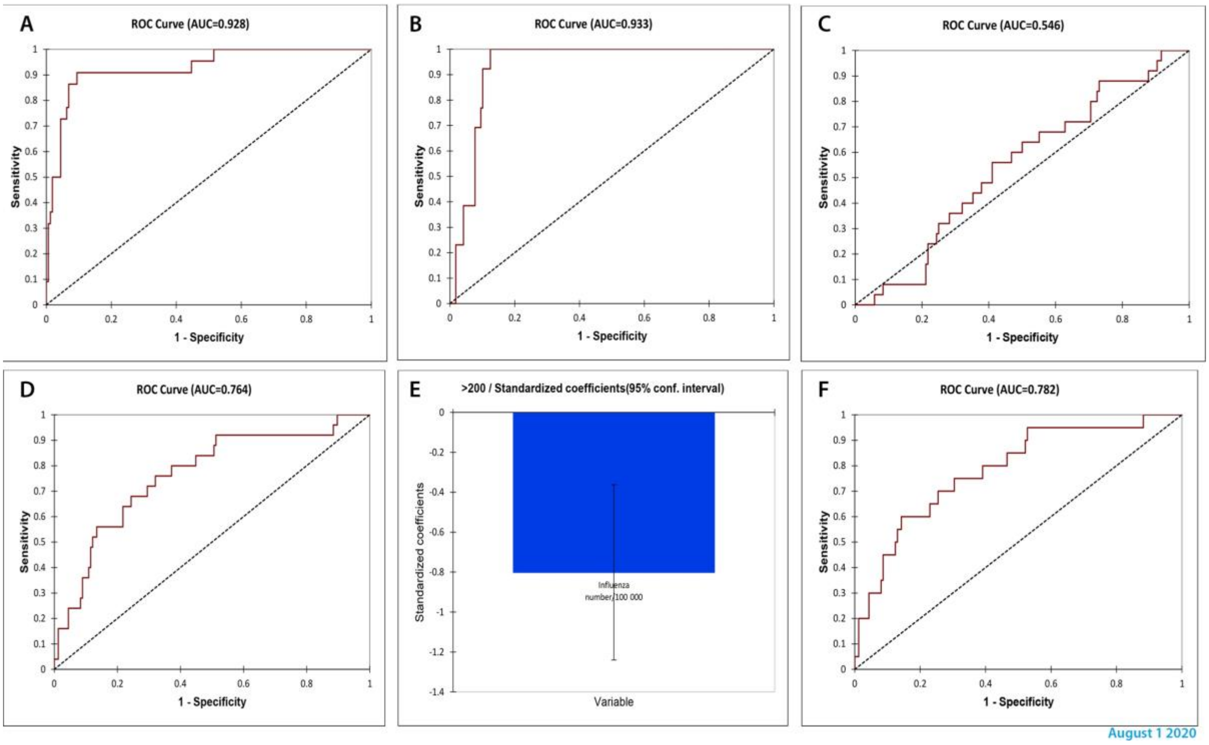

Supplement figure S10. Logistic regression analysis in a model with influenza incidence, population density and population numbers of various countries (n=182) to indicate Covid-19 mortality >200/million, and the ROC curve of the model (AUC=0.785), and morality >250/million (Panel C) and the ROC curve (AUC 0.801).

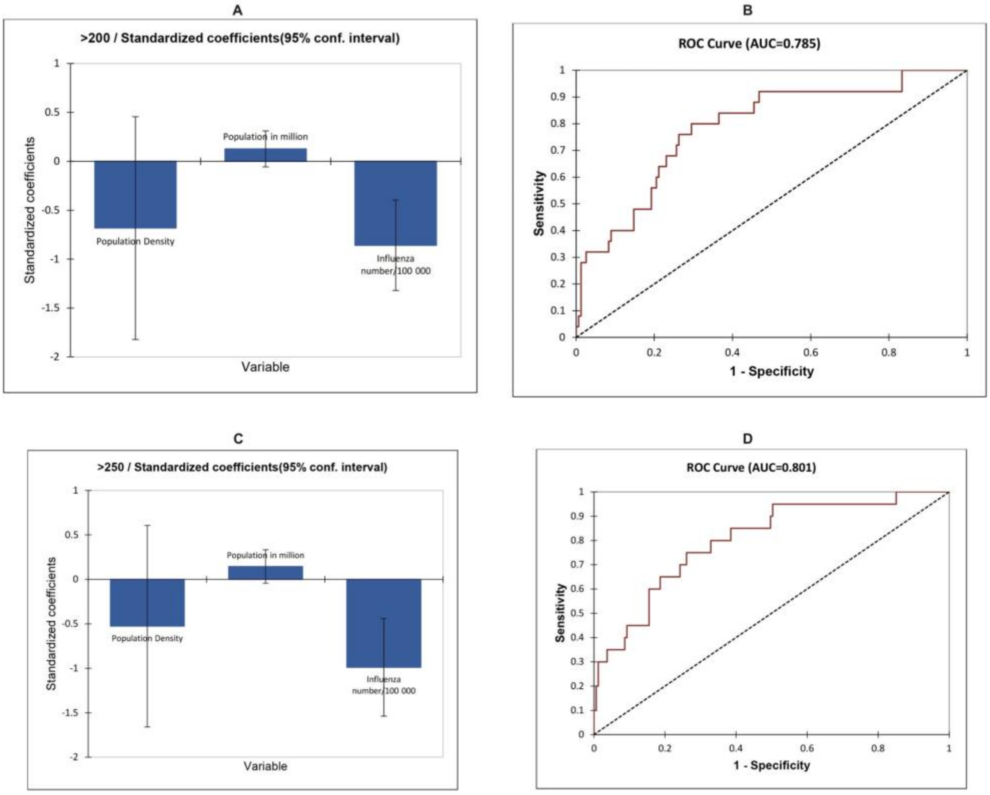

Supplement figure S11. Correlation between influenza LRI incidence*population density/100 Vs. Covid19 mortality(August 1, 2020).

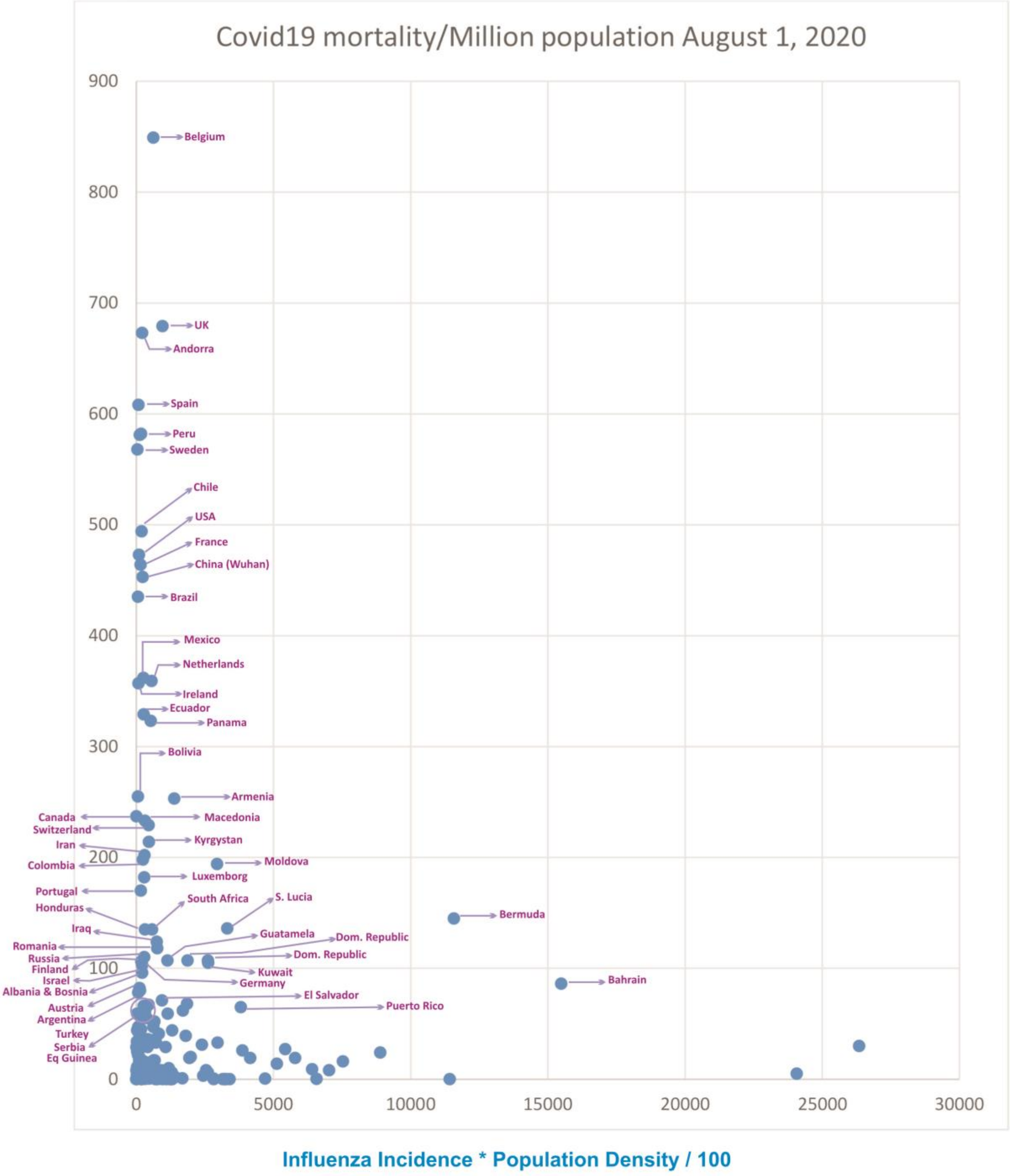

Supplement figure S12. Logistic regression analysis for influenza LRI incidence*population density and Covid19 mortality. Odd's ratio for Covid19 mortality of >200/million is -2.648; CI -4.8 to -0.488, P=0.016 and for >250/million -2.5; CI -4.78 to -0.217, P=0.03 (August1, 2020).

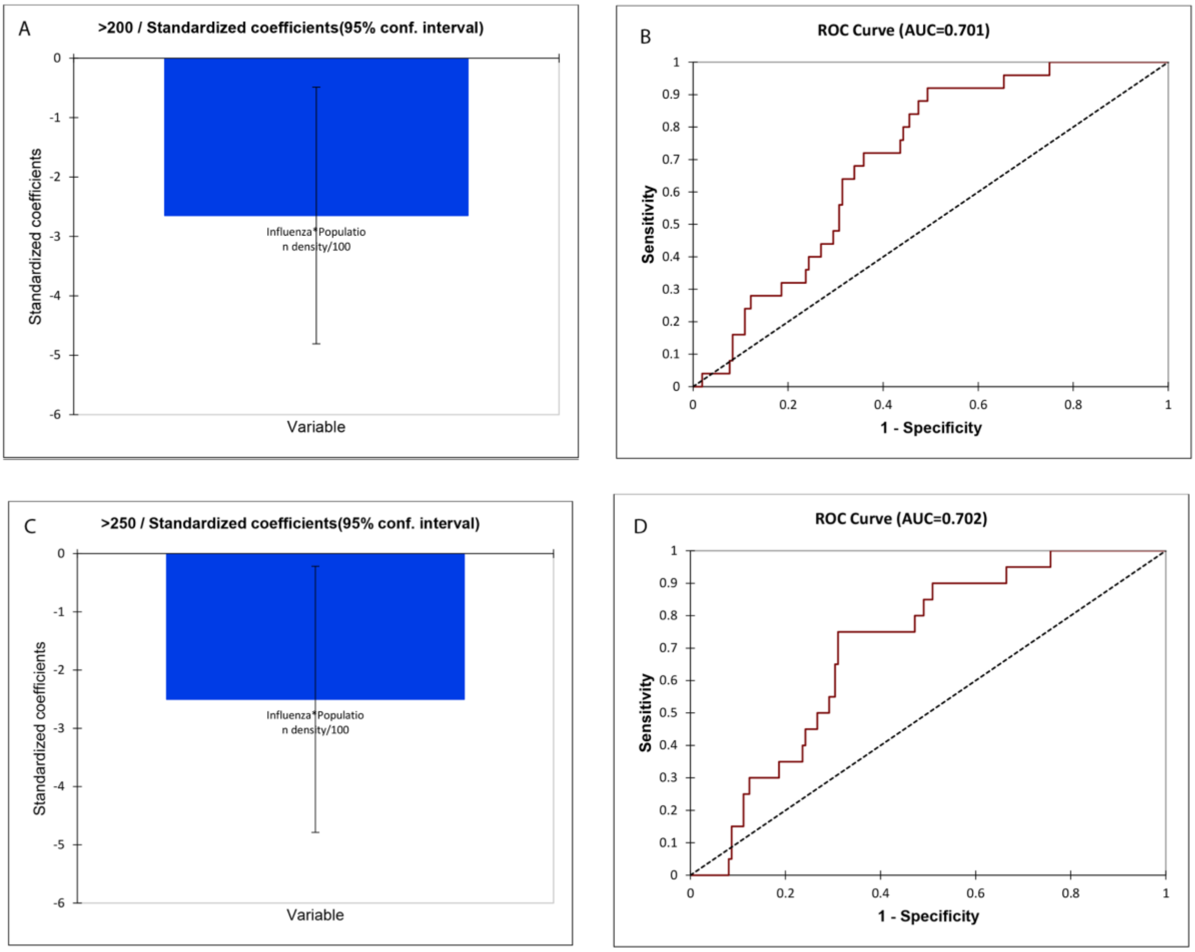

Supplement Figure S13. Logistic regression model to indicate Covid-19 deaths >250/million (Panel A), one death in <3000 people (Panel B), and mortality >200/million (Panel C) using influenza incidence, population density and population in million, August 14, 2020.

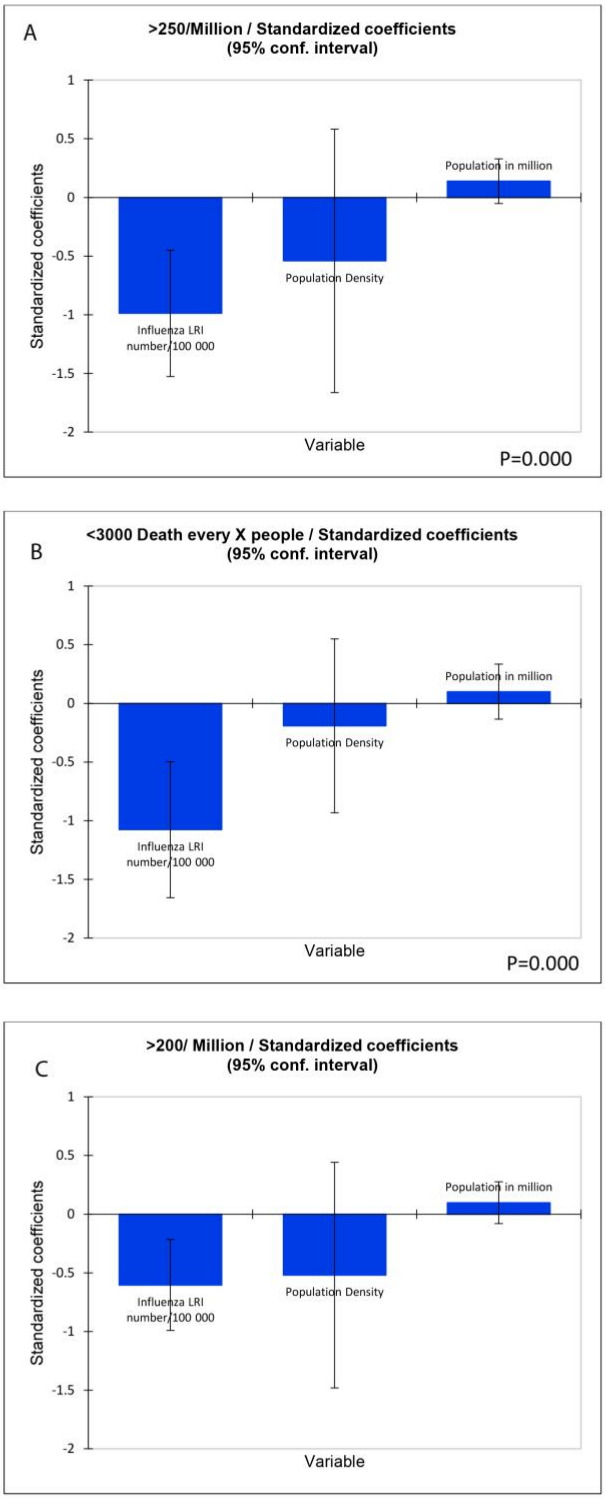

Supplement Figure S14. Logistic regression model to indicate Covid-19 deaths (Panels A and B) < 5000 death every x people, < 5000 death every x people (C and D), <3000 death every X people with influenza LRI incidence only (E and F), < 5000 death every x people (G and H), < 6000 death x people (I and J) August 15, 2020.

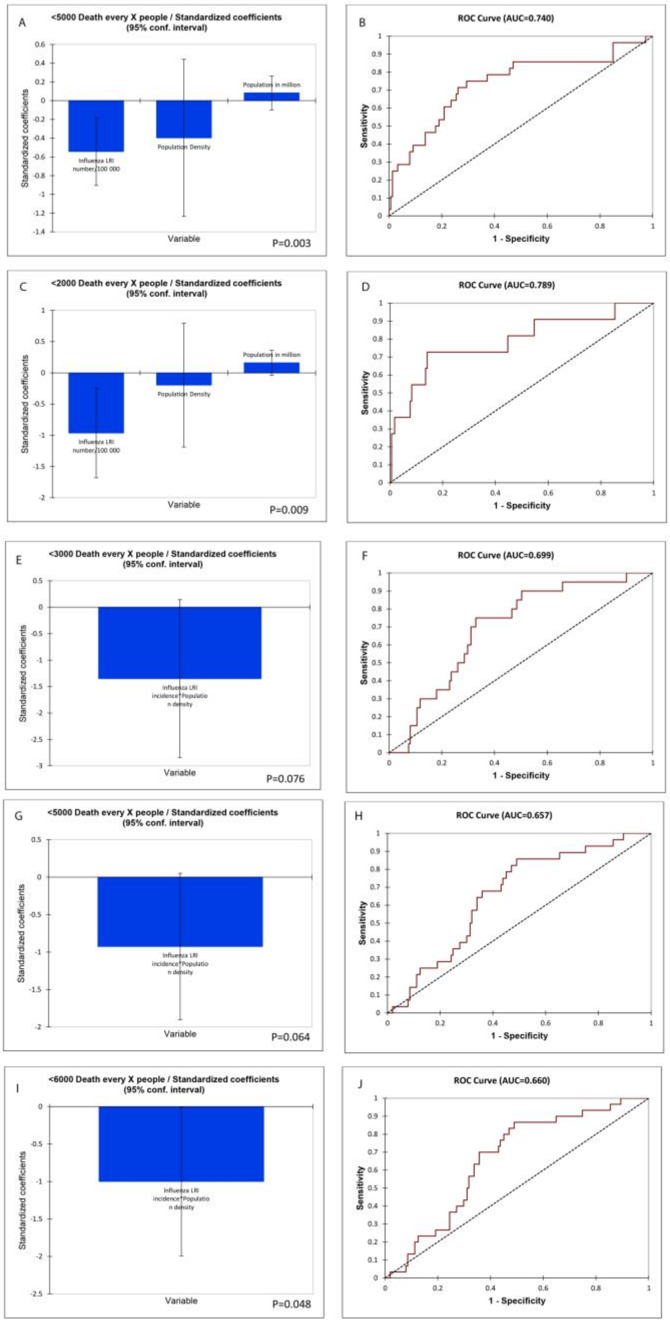

Supplement Figure S15. Logistic regression model to indicate Covid-19 deaths (A and B) >250/million, >200/million (C and D), <2000 death every X people with influenza LRI incidence parameter only (E and F), < 3000 death every x people (G and H), < 5000 death x people (I and J)and < 5000 death x people (I and J), August 16, 2020.

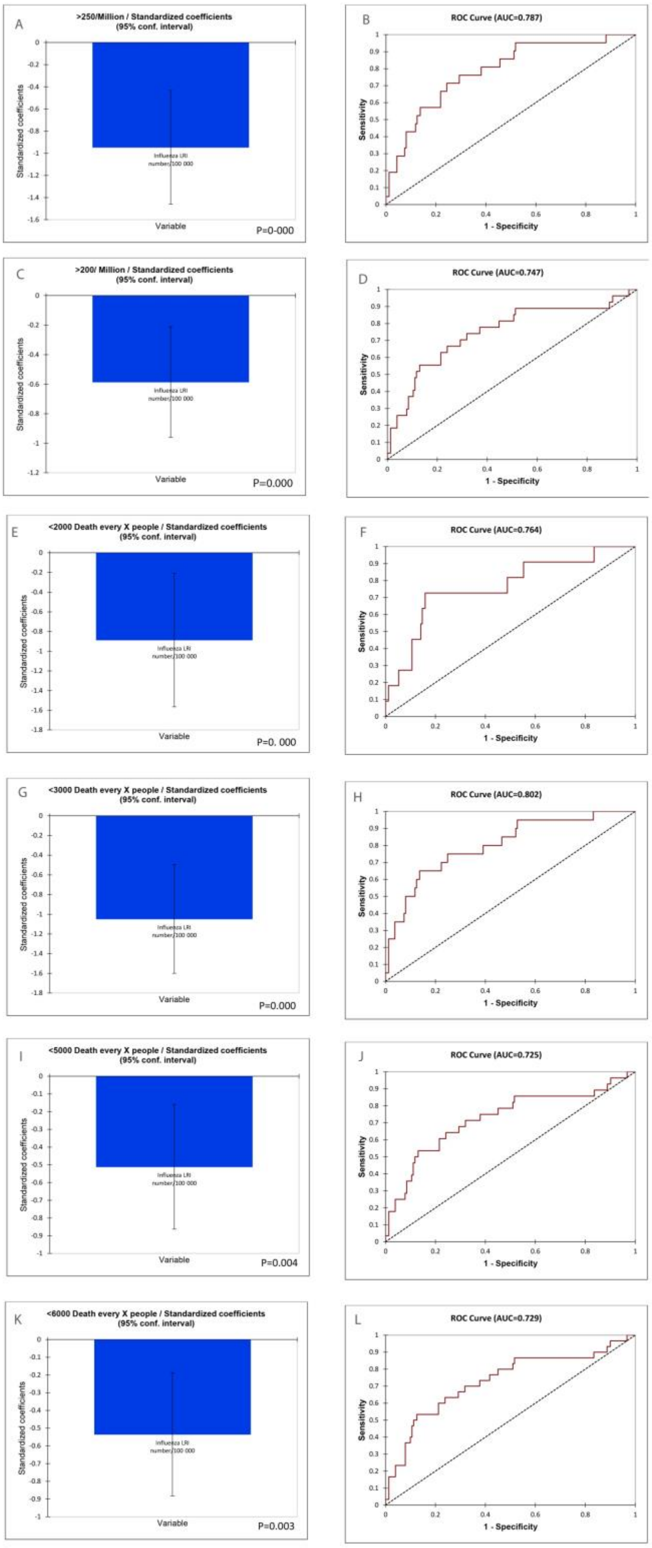

Supplement figure S16. Lower respiratory tract infection incidence in various WHO regions and Covid19 mortality (A and B, Logarithmic R2 = 0.16). Lower respiratory tract infection related deaths in various WHO regions and Covid19 mortality (C and D) (August 15, 2020).

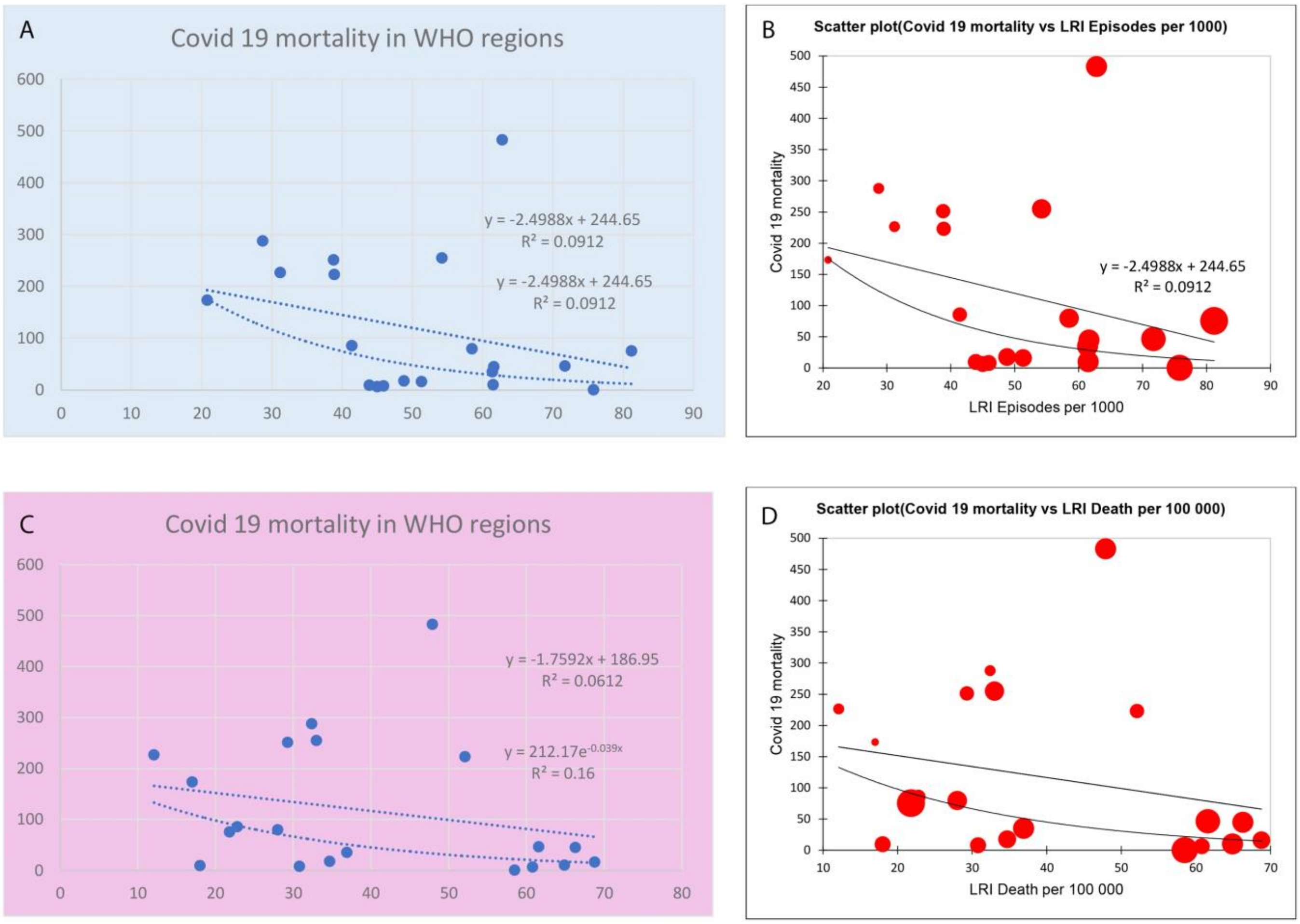

Supplemental table ST1. Covid-19 mortality and influenza Vaccination (August 14, 2020).

Country	Vaccination	Population in Million	Mortality/Million
USA	67.5	330	521
Spain	53.7	47	612
Italy	52.7	60	585
Germany	34.8	82	111
France	49.7	67	466
Iran	25	66	233
UK	72.6	81	609
Chile	64.7	18	543
Belgium	31	11.5	857
Switzerland	38	8	230
Netherlands	64	17.3	360
Canada	61.1	37.6	239
Austria	14	9	801
Portugal	60.8	10.3	174
South Korea	82.7	51.5	6
Sweden	49.4	10.2	572
Norway	34.4	5.4	48
Finland	48.4	5.5	60
Denmark	52	5.5	107
Luxembourg	37.6	0.6	196
Estonia	4.8	1.4	47
Iceland	4	0.37	29
Australia	73	24	16
New Zealand	65	4.8	4
Ireland	57.6	4.9	359
Hungary	26.8	9.7	63
Israel	58.2	8.7	74
Lithuania	13.4	2.8	30
Czech Republic	20.3	10.5	37
Latvia	7.7	2	17
Serbia	11	7	77
Slovak republic	13	5.4	6
Turkey	7	82	71
Slovenia	11.8	2.1	62

Supplement S17. Case- fatality ratio and influenza LRI incidence parameter. Logistic regression analysis including parameters - influenza*population density, population in million, Influenza LRI incidence and population density to indicate the Covid-19 mortality/cases (case-fatality) rate >5, >4 and >3 % (Panels A, B and C; August 23, 2020).

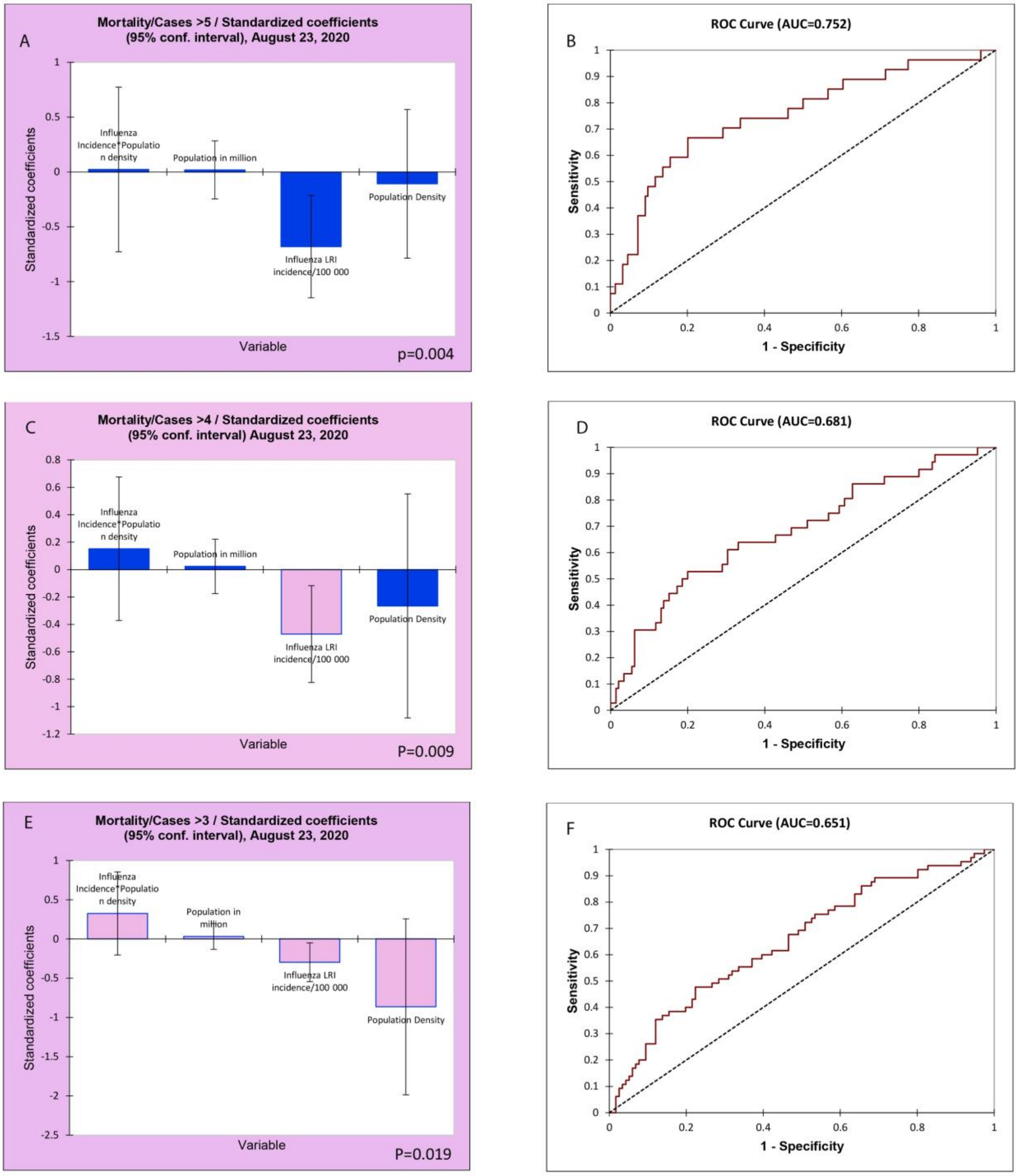

Supplement S18. Case-fatality ratio and influenza LRI incidence parameter. Logistic regression analysis with influenza LRI parameter to indicate the Covid-19 mortality/cases (case-fatality) rate >3, >4 and >5 percent (Panels A, B and C; August 23,2020).

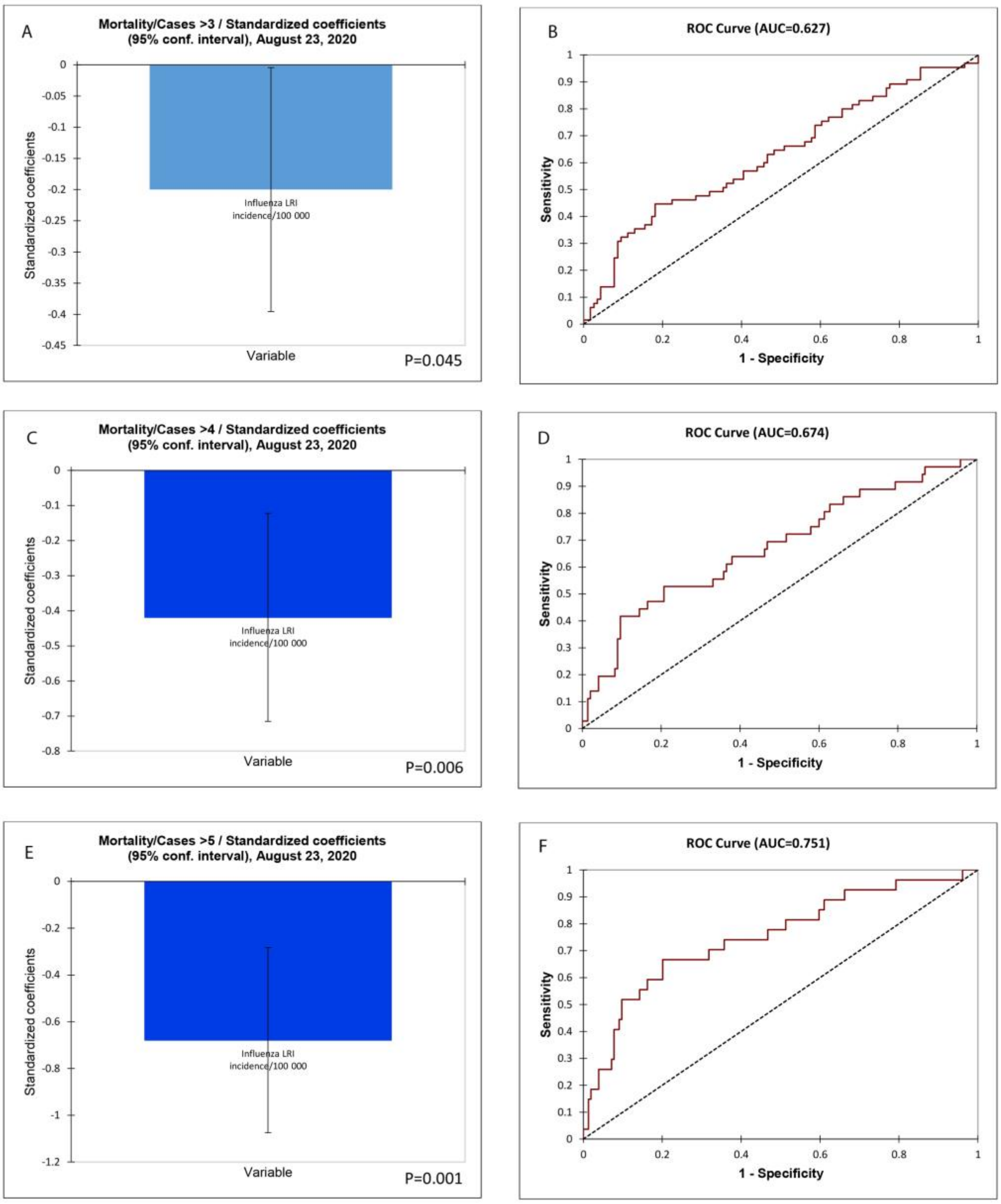

Supplement figure S19: Logistic regression with Covid-19 mortality/cases and influenza LRI*population density (Panel A and B) to indicate Covid-19 mortality/cases rate >5 percent, and Covid19 mortality>250/million. Panel C shows Influenza LRI parameter and Covid19 mortality>250/million (August 23, 2020).

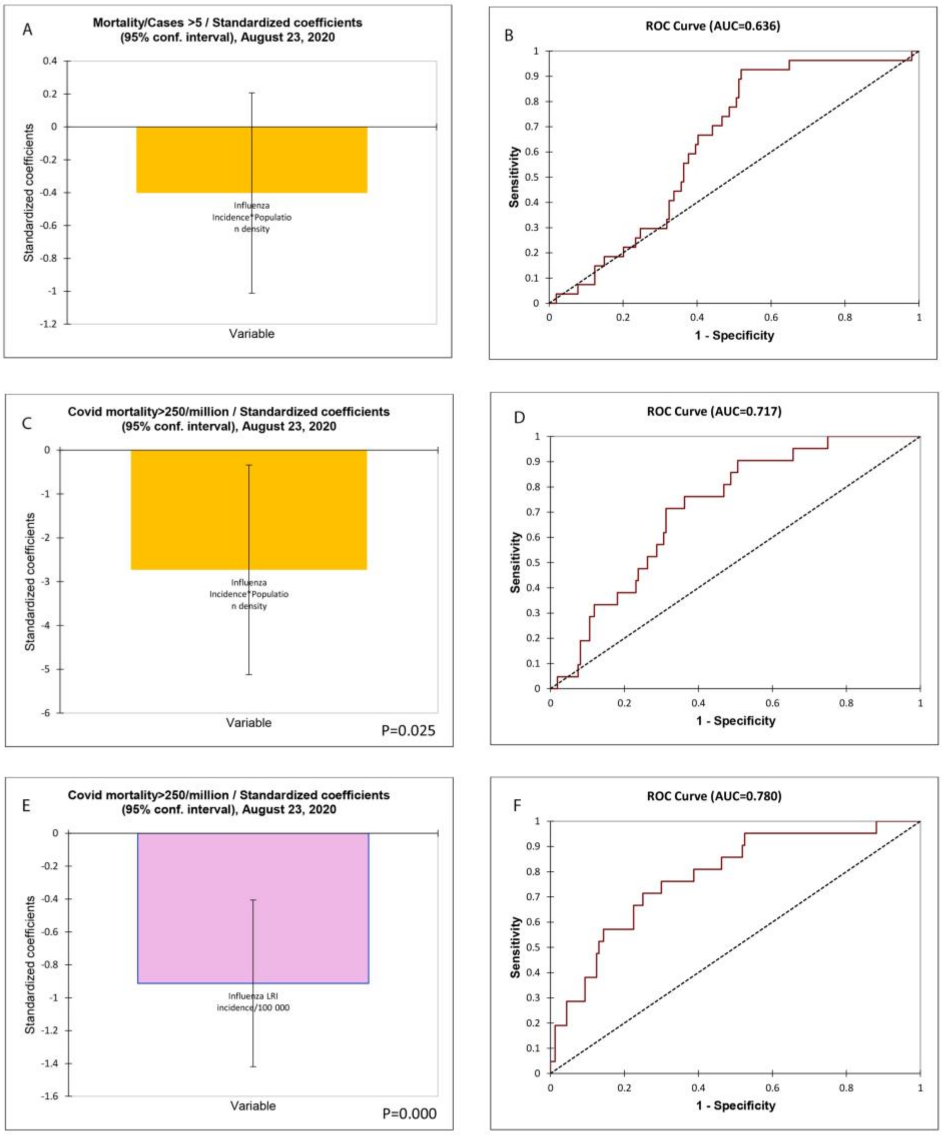

Discussion (Supplement)

The latest US military figures show a Covid19 case-fatality rate of 82/ 55 147 (0.15%) and in the veteran's affairs group 2935/ 52 089 (5.6%). The US military's influenza vaccination rate is near 100% and the mean age is 35 years. The mean age of the population in the veteran's group is 58 years, and 37% of the veteran's affairs group is ≥ 65 years. Among health care workers in the US, the vaccination percent is 81%, and the current Covid19 case-fatality rate is (670/149 195) 0.45%.

South Korea (82 to 86%), Australia 73%, New-Zealand 68%, Israel 58%, Greece 56%, Finland 49%, have higher vaccination rates and have less mortality i.e., 6/ million, 20/ million, 4/million, 91, 23 and 60/ million respectively. With a vaccination of 50% in the elderly, Japan has a mortality rate of 9/ million though not discussed in the study. Hong Kong has a vaccination rate of 50% in population 65 to 74 years and 70% in the age group ≥ 75 years, and the current Covid-19 mortality is 10/million (August 24, 2020). Greece has a lower influenza LRI incidence of 142/100 000 (CI 101 to 190/ 100 000) and it can have an increase in mortality in the future. Germany has an influenza vaccination rate of 34%, and mortality is 111/million, and Austria 15% vaccinations and death at present is 81/million. In Germany and Austria, the Covid-19 mortality is less due to strict lockdown measures, efficient house-to-house supervision and testing, and treatment. Otherwise, these mortality numbers would have been higher. The low mortality is not because of the small vaccination percentage. Also, the migrant group population is high in Germany, about 15% who tend to carry more influenza infections. Also, in the east part of Germany, where the flu vaccination ratio and influenza infections including avian influenza are higher (supplement reference 3), the Covid-19 mortality is less. In Bavaria, Baden Wurttemberg, and North Rhine-Westphalia, the Covid-19 mortality was higher. In the US and the UK, death is high, and vaccination rates are high ≥ 65 years is 65 to 68% and 72%, respectively. The addition of unregistered migrants, age>50 years, travellers, and those without insurance will bring down the overall vaccination rates substantially, and the influenza LRI infection incidence (US 287 CI 201 to 391 and UK 222 CI 158 to 297 /100 000) is low in these two countries. Especially in regions located on the lower side of the confidence intervals, the susceptibility to Covid-19 mortality is higher. In the opinion of the author, if the vaccination rates were less in these countries, the Covid-19 mortality would have been much higher than the current existing numbers.

Influenza's lower respiratory tract infections are high in South Asian and Southeast Asian countries, Russia, eastern Europe, Africa, and the Middle East. This higher influenza incidence would mean a direct vaccination by influenza and not necessarily by injections.

Hence, countries like India and its neighbours, South East Asian countries, Russia, Africa and eastern Europe have low mortality and have high passive immunity for influenza. For comparison, the influenza LRI incidence rates in Spain are about 91/100 000, Italy 63/ 100 000, Germany 120/ 100 000, UK 222/ 100 000, US 287/100 000. In eastern Europe it is about 2000/100 000, Vietnam 3700/100 000, India 1000/ 100 000 in general. South American countries it is about 500/ 100 000, and in many South and Central American countries, it is less than 500/100 000.

The GBD data could underestimate the influenza incidence to a certain extent. Also, in many Asian and African countries, this underestimate factor in influenza LRI incidence could be about 3 to 5 times higher, and the influenza LRI episodes are not well documented. Also, in these countries, due to the cultural and social structure, the elderly living at care homes are very less unlike the west, and the mortality of which is not well documented against Covid-19 dying; and in the later, the estimated correction factor is 30% which could lead to higher Covid19 deaths. People in the regions living in the lower confidence intervals in these Influenza LRI incidence values, are susceptible to more COVID 19 mortality.

Population density, Population numbers and Influenza LRI incidence

In a logistic regression analysis with population density, population numbers of various countries, and influenza incidence/100 000 of various countries (n=182) and Covid-19 mortality> 200 and > 250/million, reduced influenza incidence was associated with higher mortality (Supplement figure S10). Interestingly, lower population density and higher population numbers tended higher Covid-19 mortality though the results were not statistically significant. ROC curve fit for the logistic regression models had AUC of 0.78 and 0.801 for the model to predict Covid-19 mortality >200/million and >250/million, respectively.

Influenza * population density

The parameter population density*Influenza LRI incidence was studied as an indicator of Covid-19 mortality. Supplementary figure S11 shows the countries with low product values

which are associated with high mortality. In the logistic regression models (Supplementary figure S12) population density* influenza LRI incidence had an odd's ratio of -0.5 (CI -1.47 to +0.44, P=0.29) for Covid-19 mortality (>200/million) and the odd's ratio to indicate Covid-19 death of >250/million was -2.7 (CI -4.86 to -0.53, P<0.0001, August 1, 2020). Hence, countries or regions with low influenza incidence and especially with low population density are susceptible to higher Covid-19 mortality. Therefore, the influenza vaccine will be a useful preventive measure. Low influenza LRI incidence and low population density combination is especially seen in all western European countries, New Zealand, Poland, South America, central Latin America, Scandinavia, Brunei, and some of the middle-east countries. This susceptibility would be more seen when lockdown measures are eased due to economic purposes.

Peru, Chile, Bolivia, Ecuador are showing a rise in Covid-19 mortality. These countries have an influenza LRI incidence of about 500 to 700/ 100 000 with wide confidence intervals in these values. Also, the population density in these countries is less. Even looking at the influenza samples received and tested benefits, in the last two years, there was a declining trend in the samples tested, which indicates a lesser influenza burden compared to the previous four years.

In the logistic regression models including the parameters influenza LRI incidence, Population density and total population of various countries (n=182), lower influenza LRI incidence has significant contribution in reduction in Covid19 mortality Supplement figures S13, S14 and S15 (August 13 to 15, 2020). Supplement figures S15 and S16 shows a lower influenza LRI incidence is associated with higher Covid-19 mortality which is reflected by higher odds ratio for Covid-19 mortality >250/million, and mortality in every < 2000 or 3000 x people.

Population density has a tendency to be associated with mortality i.e., lower population density associated with higher mortality but statistically the observations were not significant (Supplement figure S14). A higher population numbers were associated with lesser mortality though not statistically significant. In some countries like Greenland, Iceland, Finland, Norway, Chad, Mauritania, Mongolia, Australia, and New Zealand, with very low population density, the Covid-19 mortality is less. This would be a phenomenon of physiological social-distancing due to significantly less population density, reducing the incidence of Covid-19 in these countries. However, in the long term, the Covid-19 severity would be high.

Lower respiratory tract infections in WHO regions and Covid19 mortality

The lower respiratory tract infections data in various WHO regions (GBD data) with mean Covid-19 mortality across diverse WHO regions showed a reduction in Covid-19 deaths in the WHO regions associated with higher incidence and mortality due to lower respiratory tract infections (Supplement figure S16). In many low-income countries, the reporting of lower respiratory tract infections (LRIs) is less, and hence the LRI burden is underestimated. If a correction is performed using this under-reporting factor, the dichotomy of the LRI burden would be higher between the high-income and low-income countries. Therefore, the observed LRI burden would be higher in low-income countries. Applying this information in addition to influenza vaccination, bacteria-based vaccines like pneumococcal vaccines would also offer additive benefits. Other bacterial vaccines like BCG and streptococcus pyogenes vaccines would also be useful, requiring further evaluation.

Case-fatality rate

The higher case-fatality rate was also significantly associated with fewer values of the influenza LRI parameter. The association of lower values of influenza LRI incidence parameter is distinctly seen with increasing severity of the Covid19 case-fatality rate from 3 to 5 percent. (Supplement figures S17 to S19, August 23, 2020).

Correlation method

In the study, mortality/ vaccination was done, and both are scientifically closely related parameters and have cause and effect relationship. This graphical plotting is a sort of parabolic method of function ($y = x^2$). The parabolic nature ($y = x^2$) of the graph can be achieved by plotting the X-axis coordinates in Z-axis also, and thereby the function $Y = X*Z$ or X^2 can be achieved. The R^2 obtained by this method may not be strongly interpreted, but the tendency of the negative slope consistently means a reduction pattern. In the paper, this function was achieved by the mortality/ vaccination method.

However, significant importance with the vaccination data is not stressed in this paper as this data is available in only a few countries, and information is available only for ≥ 65 years. Still, a tendency of beneficial effects is observed. However, substantial conclusions cannot be drawn from available influenza vaccination data alone.

Supplementary references:

S1.Thair S, He Y, Hasin-Brumshtein Y, Sakaram S, Pandya R, Toh J et al. Transcriptomic Similarities and Differences in Host Response between SARS-CoV-2 and Other Viral Infections. MedRxiv. 2020; doi:10.1101/2020.06.18.20131326.

S2. Fink G, Orlova-Fink N, Schindler T, Grisi S, Ferrer A, Daubenberger C et al. Inactivated trivalent influenza vaccine is associated with lower mortality among Covid-19 patients in Brazil. 2020; doi:10.1101/2020.06.29.20142505.

S3. Globig A, Staubach C, Sauter-Louis C, Dietze K, Homeier-Bachmann T, Probst C et al. Highly Pathogenic Avian Influenza H5N8 Clade 2.3.4.4b in Germany in 2016/2017. Frontiers in Veterinary Science. 2018;4. doi.org/10.3389/fvets.2017.00240.
